# The impact of living with multiple long-term conditions (multimorbidity) on everyday life – a qualitative evidence synthesis

**DOI:** 10.1186/s12889-024-20763-8

**Published:** 2024-12-18

**Authors:** Emilia Holland, Kate Matthews, Sara Macdonald, Mark Ashworth, Lynn Laidlaw, Kelly Sum Yuet Cheung, Sebastian Stannard, Nick A. Francis, Frances S. Mair, Charlotte Gooding, Nisreen A. Alwan, Simon D. S. Fraser

**Affiliations:** 1 School of Primary Care, Population Sciences and Medical Education, Faculty of Medicine, University of Southampton, Southampton General Hospital, Tremona Road, Southampton, SO16 6YD UK; 2 General Practice & Primary Care, School of Health and Wellbeing, University of Glasgow, Glasgow, UK; 3 School of Life Course and Population Sciences, King’s College London, London, UK; 4 Patient and Public Involvement (PPI) Member, MELD-B Project, Southampton, UK; 5 Patient and Public Involvement and Engagement, University Hospital Southampton NHS Foundation Trust, Southampton, UK; 6 University Hospital Southampton NHS Foundation Trust, Southampton, UK; 7 NIHR Applied Research Collaboration Wessex, Southampton, UK

**Keywords:** Multimorbidity, Long-term conditions, Burden, Impact, Work, Lived experience

## Abstract

**Background:**

Multiple long-term conditions (MLTCs), living with two or more long-term conditions (LTCs), often termed multimorbidity, has a high and increasing prevalence globally with earlier age of onset in people living in deprived communities. A holistic understanding of the patient’s perspective of the work associated with living with MLTCs is needed. This study aimed to synthesise qualitative evidence describing the experiences of people living with MLTCs (multimorbidity) and to develop a greater understanding of the effect on people’s lives and ways in which living with MLTCs is 'burdensome' for people.

**Methods:**

Three concepts (multimorbidity, burden and lived experience) were used to develop search terms. A broad qualitative filter was applied. MEDLINE (Ovid), EMBASE (Ovid), PsycINFO (EBSCO), CINAHL (EBSCO) and the Cochrane Library were searched from January 2000-January 2023. We included studies where at least 50% of study participants were living with three or more LTCs and the lived experience of MLTCs was expressed from the patient perspective. Screening and quality assessment (CASP checklist) was undertaken by two independent researchers. Data was synthesised using an inductive approach. PPI (Patient and Public Involvement) input was included throughout.

**Results:**

Of 30,803 references identified, 46 met the inclusion criteria. 31 studies (67%) did not mention ethnicity or race of participants and socioeconomic factors were inconsistently described. Only two studies involved low- and middle-income countries (LMICs). Eight themes of work were generated: learning and adapting; accumulation and complexity; symptoms; emotions; investigation and monitoring; health service and administration; medication; and finance. The quality of studies was generally high. 41 papers had no PPI involvement reported and none had PPI contributor co-authors.

**Conclusions:**

The impact of living with MLTCs was experienced as a multifaceted and complex workload involving multiple types of work, many of which are reciprocally linked. Much of this work, and the associated impact on people, may not be apparent to healthcare staff, and current health systems and policies are poorly equipped to meet the needs of this growing population. There was a paucity of data from LMICs and insufficient information on how patient characteristics might influence experiences. Future research should involve patients as partners and focus on these evidence gaps.

**Supplementary Information:**

The online version contains supplementary material available at 10.1186/s12889-024-20763-8.

## Background

Multiple long-term conditions (MLTCs) or ‘multimorbidity’, usually defined as living with two or more long-term conditions (LTCs) where each condition is given equal importance, is distinct from co-morbidity where one condition is considered the index condition with additional co-occurring conditions [1]. MTLCs is common, has increased in prevalence over the last 20 years in many countries, and is having major impacts on health and social care systems and people’s lives [2–5]. Women generally experience a higher burden of MLTCs than men, and people from certain ethnic groups and those living with greater socioeconomic deprivation develop MLTCs earlier in life and such inequalities are increasing [6–8].

The challenges presented by various aspects of living with MLTCs have previously been characterised as ‘burden’, including symptom burden and treatment burden, which both affect wellbeing [9, 10]. Several models have been developed to capture these challenges. In 1985, Corbin and Strauss described the three lines of work model for managing chronic illness at home, incorporating ‘illness work’, ‘everyday life work’ and ‘biographical work’ [11]. The 2012 Cumulative Complexity Model described the balance between the workload of demands on people living with LTCs and their capacity to address those demands [12]. And the 2013 Burden of Treatment Theory described burden of treatment as the work associated with healthcare [13, 14].

The type and number of LTCs a patient is living with is important, and increasing LTC count is associated with higher treatment burden and symptom burden [9, 15]. Many studies have quantified, grouped and clustered MLTCs by number and type of conditions. However, some conditions are more challenging than others for patients in terms of symptoms, impacting self-management demands (burden of treatment) and health-related quality of life [13, 14, 16–19]. In addition, MLTCs usually develop across the lifecourse with their impact on people’s lives developing and changing over time [5].

The individual context of the patient’s life also affects burden. Recent evidence from South Africa, Malawi and the UK has shown that financial precarity both increases and affects the capacity to manage treatment burden, and a recent UK study recently identified high treatment burden in some people experiencing homelessness [20–23].

Many health systems are organised around single conditions and there is evidence that some people experience ‘burnout’ resulting from the demands of LTCs and their self-management tasks [24]. From the perspective of people with MLTCs, such problems may be multiplied, and a holistic understanding of the many demands of living with MLTCs from a patient perspective is therefore needed.

Several studies have explored aspects of the lived experience of MLTCs involving a variety of LTC combinations [13, 19, 25–32]. Additionally, in 2017, Rosbach and Andersen conducted a systematic review focussing on burden of treatment in patients with MLTCs [15].

The aim of this study was to synthesise published qualitative evidence describing the experiences of living with MLTCs (multimorbidity) and develop a greater understanding of the effect on people’s lives and ways in which living with MLTCs is 'burdensome' for people in order to understand the holistic experience of everyday life for people living with MLTCs. In keeping with this aim, our study was co-produced with PPI (Patient and Public Involvement) colleagues.

## Methods

This qualitative evidence synthesis was undertaken as part of the NIHR-funded Multidisciplinary Ecosystem to study Lifecourse Determinants and Prevention of Early-onset Burdensome Multimorbidity (MELD-B) study [33]. We report our search according to the ENTREQ checklist (Supplementary Table 2) [34].

Advice regarding qualitative systematic review methods was provided by subject expert SM. The protocol was registered with the International Prospective Register of Systematic Reviews (PROSPERO, Registration Number CRD42023391056) [35]. The primary research question ‘What is it like to live with MLTCs (multimorbidity) and which aspects do people living with MLTCs consider burdensome and make living with multimorbidity complex?’ was initially developed using the PerSPecTIF and SPIDER frameworks [36, 37]. Discussions with PPI colleagues led to a broadening of the study population from primary care patients to ‘people living with MLTCs because some people will be more commonly reviewed in secondary care and rarely seen in primary care. Our discussions also highlighted that ‘work ‘, opposed to ‘burden’, was a better way to frame the concepts of difficulties and challenges associated with living with MLTCs. The word ‘work’ will therefore be used in preference to ‘burden’ where relevant in the manuscript. The secondary research question was ‘Was there any PPI input into the papers identified by this review?’.

### Inclusion and exclusion criteria

Inclusion and exclusion criteria are shown in Table 1.

**Table 1:** Inclusion and exclusion criteria

Inclusion Criteria	Exclusion Criteria
Studies involving papers where at least 50% of participants were living with three or more LTCs (the remaining participants were living with at least one LTC)	Studies involving papers where fewer than 50% of participants were living with three or more LTCs
A focus on multimorbidity (not a focus on one or two conditions with comorbidity)	Studies focussing on one or two clear index conditions and comorbidities (This allowed a greater focus on multimorbidity rather than co-morbidity and was also a pragmatic decision due to the very high number of studies identified by the criteria of two or more LTCs)
Studies exploring lived experience of MLTCs from the point of view of patients	Not from the patient perspective
Qualitative studies (primary research or qualitative syntheses) and mixed methods studies with a relevant qualitative component	Quantitative studies (except mixed methods studies with a substantial qualitative component)
All settings including home and other community settings (including private, rented, social housing, care home, prisons, homeless) and clinical settings (primary care, secondary care, intermediate care, etc.)	Studies including children
	Not in English
	Conference abstract (no full text article)
	Duplicate (the same study with more than one record in Rayyan)
	Studies with a focus on medicines
	Studies with a focus on self-management (helping people manage better)
	Studies with a focus on the use of technology (e.g. patients’ views on telehealth)
	Studies with a focus on interventions

We included qualitative studies (primary research or qualitative syntheses) and mixed methods studies with a substantial qualitative component where at least 50% of participants were living with three or more LTCs. This allowed a greater focus on multimorbidity rather than co-morbidity and was also a pragmatic decision due to the very high number of studies identified by the criteria of two or more LTCs. We excluded ‘comorbidity’ studies due to the distinction of multimorbidity having no index disease and “all morbidities…regarded of equal importance” [38, 39]. In view of our focus on the lived experience of multimorbidity we also excluded studies principally focusing on interventions, medicines, technology and self-management.

### Search strategy

The search strategy was developed in consultation with Patient and Public Involvement (PPI) members, subject matter experts (SF, SM, FM, MA, NF) and with advice from engagement librarians at the University of Southampton. Search terms were developed as three concepts (‘multimorbidity’, ‘burden’ and ‘lived experience’) with a qualitative filter, each with a string of terms and relevant MeSH terms, and were developed from a review of grey literature reports, published searches, PhD/MD theses and an online thesaurus [40]. The search was refined in study team meetings. The full searches are available in the Supplementary Table 1.

The searches were conducted in January 2023 and the date range was restricted to 1st January 2000 onwards for pragmatic reasons (the very high number of studies). The date range for the term ‘comorbidity’ was restricted from 2000–2018 in MEDLINE and Embase as this term was used prior to the introduction of the Medical Subject Headings (MeSH) terms ‘multiple chronic conditions’ and ‘multiple long-term conditions’ by the National Library of Medicine (NIH) in 2017 and 2018 respectively [41–43].

The following databases were searched: MEDLINE (Ovid), EMBASE (Ovid), PsycINFO (EBSCO), PsycArticles (EBSCO), CINAHL (EBSCO) and the Cochrane Library, and references were stored in EndNote. The Journal of Multimorbidity and Comorbidity was manually searched for additional references and we undertook reference list searching from included articles.

### Screening

Double screening of the title/abstract of all studies was conducted using Rayyan software (apart from screening of Cochrane Library studies which was undertaken in Excel for pragmatic reasons) [44]. Rayyan records identified for full text screening were exported into Excel. We searched for full text PDFs for all articles. Any articles which were identified as being a conference abstract were excluded at this point. EH created an Excel template for full text screening. EH read and assessed all papers (blinded to the results of the second screeners). A team of screeners (KM, KSYC, LL, MA, SF) were emailed the screening spreadsheet and independently assessed a subset of the full texts and completed the Excel template. Once all full text reviews were returned, EH compared the two decisions. Any discrepancies were adjudicated by senior author SF. Studies were not excluded based on quality.

### Quality appraisal of included studies

Two researchers (EH and one of KSYC, SS, MA, CG, SF) independently undertook the quality assessment of included studies (blinded) using the Critical Appraisal Skills Programme checklist for qualitative research [45, 46]. In any cases where there was initial disagreement between the two assessors on an aspect of study quality then a conservative approach was adopted and the lower quality category was chosen.

### Data extraction

Information on the author, year of publication, location, study design, number of participants, participants’ age, sex, ethnicity and socioeconomic status, and number of LTCs were extracted from the methods and results section of included papers. Individual studies were checked for duplicate populations and included reviews were checked to see whether they incorporated individual studies that were included separately in our study. If this was found to be the case, the individual studies were not excluded, but the potential for double representation of those studies was noted and considered in the analysis process. Paper PDFs were converted into word documents and imported into NVivo [47].

### Data analysis

The method of synthesis was determined by the data, rather than apriori, as is considered best practice [46, 48]. Line by line coding was undertaken in NVivo for all studies by EH. All text relating to burden in Results and Discussion sections of papers were coded, except where information was not from the patient perspective, for example in studies who also interviewed spouses, caregivers and healthcare providers.

A second coder (SF) manually coded 10% (five papers) and coding was compared for agreement, with no new codes being generated. Regular lengthy and active discussions took place between EH, SF and subject expert SM over the course of the analysis. We followed ‘RETREAT’ guidance to choose methodology and undertook thematic synthesis [49]. We initiated analysis by coding to several broad burden themes relating to symptoms and treatment burden. Such themes were drawn from our collective research experience in this area. These broad themes provided an initial analytic architecture.

Our analysis then proceeded in an iterative manner, adding more depth, themes and sub-themes, with the relative importance of each shifting as we progressed. We initially developed descriptive themes. These themes gave a description of the experience of living with MLTCs, by reference to how people described their experiences in the papers. Our analytical themes provide an interpretation of what ‘burdensomeness’ meant, by considering the experiences together with a broader understanding of the impact. These analytical themes were informed by our knowledge of the lack of a holistic approach for people living with MLTCs, and our insights that healthcare and self-management are themselves sources of work for patients. Concepts were derived either from a single code or a group of related codes within a theme. These were too numerous to be subthemes.

A ‘constant comparison’ approach to discussions was taken whereby codes and emerging themes were repeatedly discussed and iterated over several months within the research team (including PPI coauthor LL, and subject experts FM, MA, NF, SF, SM), the wider MELD-B team, and with the wider MELD-B PPI Advisory Board to check for relevance and understanding.

### GRADE-CERQual assessment of study findings

The GRADE-CERQual (Confidence in Evidence from Reviews of Qualitative research) assessment approach was carried by two researchers together (EH, SF) [50]. This process assesses the key issues for included papers in a qualitative evidence synthesis around four concepts: methodological limitations, coherence, data adequacy and relevance, to assess confidence of the evidence from the review [50].

### Reflexivity

EH, SF FM, NF and MA have experience of caring for people with MLTCs in general practice. FM, SM, MA and SF are academic MLTCs subject experts. LL is a PPI member with personal experience of living with MLTCs. KM is a junior hospital doctor. KSYC is a PPI officer and researcher. SS is a post-doctoral researcher with expertise in lifecourse epidemiology. CG is a public health registrar and previous physiotherapist. NA has a clinical background and is a subject expert in epidemiology, early life and long Covid.

We acknowledge that our authors’ a priori experience meant that we could not be value free when conducting this study. We are a primary care centric team with a strong representation from medicine. Our interpretation of findings may have been influenced by many years of experience consulting patients and taking a medical perspective. However, we feel this was balanced by input from PPI and social science within the team.

Regular reflexive practice occurred during team meetings (including PPI), where our discussions were shaped by discussion of our prior academic experience and our lived experience of MLTCs. These discussions led to our adoption of the preferred terminology of ‘work’ rather than ‘burden’, and also allowed us to discuss whether emotions could be considered as ‘work’. Our public contributors additionally suggested the inclusion of a GRIPP2 form and wrote the first draft.

With respect to the analysis, we approached the data with a previous understanding of burden, for example treatment burden and symptom burden. We were also aware of some of the issues for people with lived experience of MLTCs and we had also gained early further insight from the title/abstract and full text screening process. We also accept that all members of our team will have been influenced by our familiarity with different conditions, particularly those which we are living with, those of family members and those where we are ‘subject experts’. Our initial descriptive themes (how people described their experiences in the papers) were developed into analytical themes by considering these experiences together with a broader understanding of the impact. These themes were shaped by discussion with those with lived experience of MLTCs.

When writing up our work we did not give any one theme more importance than another. All members of the team were invited to give feedback on the paper drafts, and all comments were given equal consideration.

### Patient and Public Involvement (PPI)

PPI input was a strength of this study as it allowed us to fully embrace the interpretive nature of an evidence synthesis—we coded the data and PPI contributors were able to add real world interpretation to what these codes might mean. This was emphasised by the co-presentation (researcher and public contributor) of our work which helped to powerfully convey our work.

LL co-developed the research questions, search strategy and synthesis method, helped to screen and assess the quality of papers, discussed and analysed emerging themes, and co-authored our research paper. The extent of patient and public involvement in the included studies was suggested and explored by LL and a GRIPP2 reporting checklist has been cowritten [51].

## Results

**Fig. 1:**
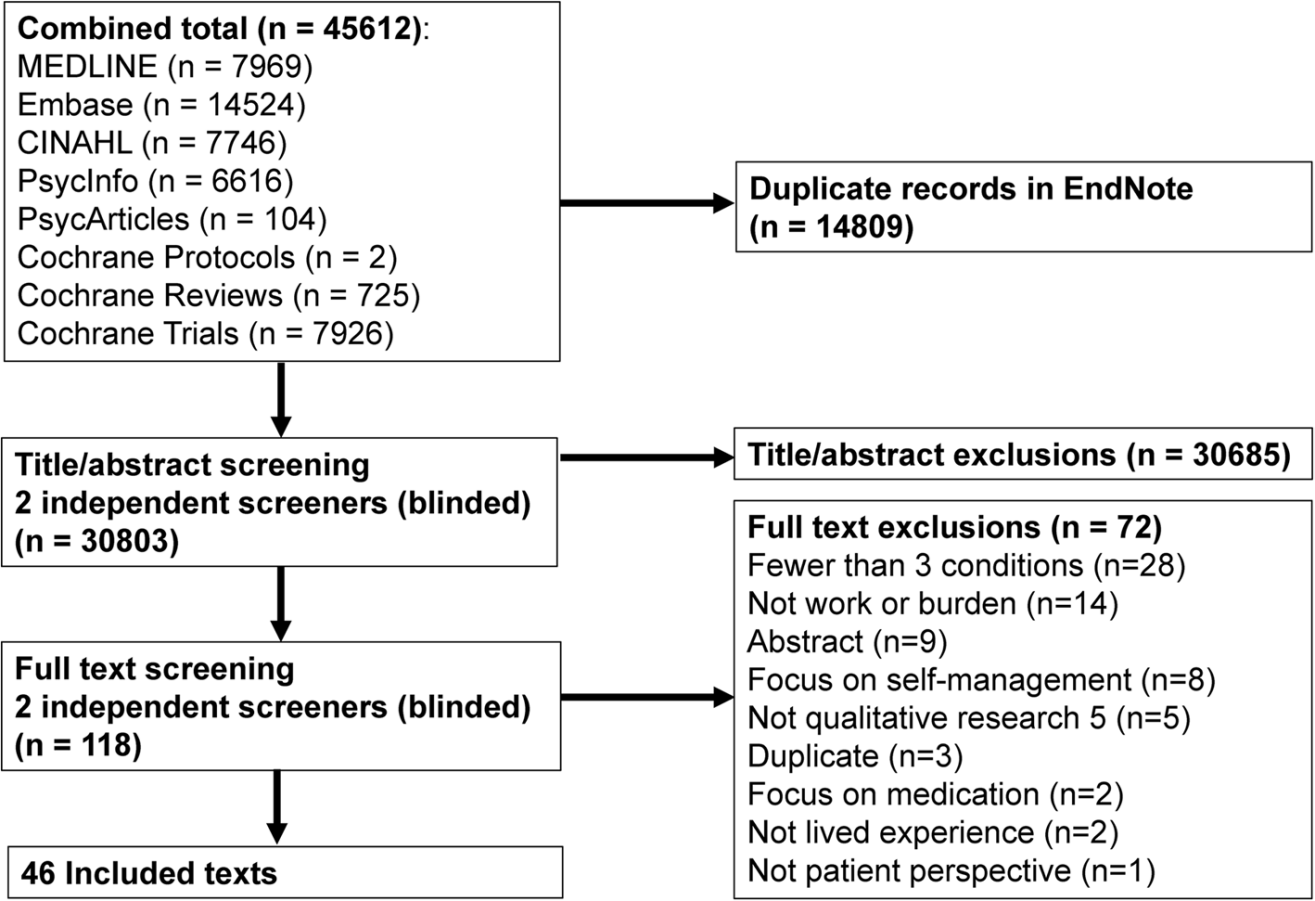
Flow diagram of study identification process

The search identified a total of 30,803 unique studies. 30,685 were excluded by title/abstract screening (exclusion reasons not recorded). 72 of the remaining 118 studies were excluded by full text screening leaving 46 included in the review (Fig. 1) [25, 29, 31, 52–94].

Exclusion reasons for full text screening were as follows: studies where fewer than 50% of participants were living with three or more LTCs (28), study not focussed on work or burden (14), conference abstract (no full text article) (9), focus on self-management (8), no substantial qualitative component (5), duplicate (the same study with more than one record in Rayyan) (3), focus on medication (2), study not focussed on lived experience (2), not from the patient perspective (1).

### Characteristics of included studies

The characteristics of included studies are shown in Table 2.

**Table 2:** Characteristics of Included Studies

**Study first author**	**Year of publication**	**Location**	**Data collection method**	**Number of participants**	**Age range of participants (years)**	**Mean age (unless otherwise specified) and SD if specified**	**Sex/Gender of participants**	**Ethnicity and/or Race of participants**	**Socioeconomic status of participants (SES)**	**Number of conditions (range unless otherwise specified)**
Aberg	2020	Sweden	Individual interviews (lifeworld)	34	74 to 96	86 (no SD specified)	11 men, 23 women	Not specified	SES not addressed	3 to 16
Ancker	2015	USA	Semi-structured interviews with patients and health care providers	22 patients	37 to 89	64 (no SD specified)	11 men, 11 women	7 black (no further details)	Considered at individual level (medical insurance details)	Mean 3.5 (SD 1.5)
Bardach	2011	USA	Two in-depth interviews, each with open-ended, semi-structured and structured questionnaires	41	51 to 77	63 (no SD specified)	12 men, 29 women	41 white (100%)	Considered at individual level (education, income, current financial status, insurance type and work status)	2 to 10, mean 4.68 (no SD specified)
Bayliss	2003	USA	Semi-structured personal interviews (free listing)	16	31 to 70 +	Not specified	3 male, 13 female	16 white (100%)	Considered at individual level (education, income and health insurance)	3 to 7, mean 4.3 (no SD specified),
Bissenbakker	2022	Denmark	Semi-structured interviews	15	39 to 84	Not specified	10 male, 5 female (calculated from table)	Not specified	Considered at individual level (education and occupation)	2 to 8 (calculated from table)
Bower	2012	UK	Face to face semi-structured interviews	28	39 to 89	Median 66 (no IQR specified)	16 male, 12 female	Not specified	SES not addressed	2 to 10, mean 4 (no SD specified)
Cheng	2019	China	Demographic questionnaire and semi‐structured face‐to‐face interviews	14	32 to 75	Not specified	8 male, 6 female	14 Chinese (100%)	Considered at an individual level (employment and educational level)	More than half (57%) had three chronic conditions
Clarke—You learn to live [59]	2013	Canada	In-depth interviews	35 (duplicate population)	73 to 91	Men 78.6, women 80.3 (no SD specified)	16 men, 19 women	Not specified	Considered at an individual level (income, educational attainment, work history)	3 to 14, mean 6 (no SD specified)
Clarke—Constructing the moral body [60]	2013	Canada	In-depth interviews	35 (duplicate population)	73 to 91	Men 78.6, women 80.3 (no SD specified)	16 men, 19 women	Not specified	Considered at an individual level (education and household income)	3 to 14, mean 6 (no SD specified)
Clarke	2008	Canada	In-depth interviews	20	68 to 88	Not specified	10 men, 10 women	Not specified	Considered at individual level (educational attainment and income) Diverse	5 to 18, mean 10 (no SD specified)
Coventry	2015	(*n* = 19 studies) USA 10, UK 6, Canada 1, Netherlands 1, Germany 1	Systematic review of qualitative studies (semi-structured interviews 13, focus groups 5, a combination of both methods 1)	Not specified (systematic review)	30 to 96	Not specified	The majority of studies, except two, recruited predominately more women	8 studies did not report ethnicity. In 6 studies the majority of participants were White participants, and Black participants; 2 studies recruited predominantly participants from black and ethnic minority communities	Considered at area level (patients living in areas of high deprivation / affluent population)	(*n* = 18 studies) 11 studies ≥ 2 LTCs, 4 studies ≥ 3, 1 study ≥ 4, 1 study ≥ 5 1 study measured multimorbidity by grouping high users of medical services
Daker-White	2018	UK	Triangulation of data from electronic health records (EHRs), observation of primary care consultations and annual interviews with patients, (informal) care providers and GPs (patient interviews—annual interviews and after primary care consultations (informal carers could also present or interviewed separately), annual interviews with GPs and/or practice nurses)	26 (one patient dropped out, data excluded)	65 to 75 +	Not specified	11 male, 14 female	Not specified	Considered at small area level (Index of Multiple Deprivation Decile)	2 to 6 or more (no mean or SD specified)
Duguay	2014	Canada	Two semi-structured interviews and collection of additional information from the family physician about the chronic diseases of participants	11	37 to 66	58.1 (no SD specified)	Male 64%, female 36%	Not specified	Considered at individual level (education and annual income)	5 to 11, mean 7 (no SD specified)
Eckerblad	2015	Sweden	Semi-structured interviews	20 (duplicate population)	79 to 89	84 (SD 2.9)	4 men, 16 women	Not specified	SES not addressed (all participants from a similar background)	Mean 12 (SD 5.3) prevalent symptoms, total symptom burden score median 0.96 (range 0.31 to 2.27)
Eckerblad	2020	Sweden	Face to face interviews	20 (duplicate population)	79 to 89	84 (SD 2.9)	4 men, 16 women	Not specified	SES not addressed	Mean 12 (SD 5.3) prevalent symptoms, total symptom burden score median 0.96 (range 0.31 to 2.27)
Etkind	2022	(*n* = 44 articles relating to 40 studies) Europe 23, USA 17, Australasia 4	Systematic review and thematic synthesis (Interview design 27, focus groups 7, other designs including ethnography, observation and mixed-methods 10)	460 (patient perspective)	Not specified (systematic review)	Not specified	Not specified	Not specified	SES not addressed (paper noted all articles were from high income western countries)	Not specified (systematic review)
Eton	2012	USA	Semi-structured interviews	32 (subset of the 50 participants in Eton 2015)	26 to 85	Median 59.5 (no IQR specified)	12 male, 20 female	31 White (97%), 1 African-American (3%)	Considered at individual level (education and employment details) Lack of socioeconomic diversity noted	Median 5 (range 1 to 16)
Eton	2015	USA	Semi-structured interviews and focus groups	50 (Interviews): 32 from Mayo Clinic (MC, same population as Eton 2012), 18 from Hennepin County Medical Center (HCMC) 25 (Focus groups): 12 from MC 13 from HCMC	25 to 85 (interviews) 47 to 87 (focus groups)	Interviews: MC median 59.5, HCMC median 50.5; (no IQR specified) Focus groups: not specified	Interviews: MC 12 male, 20 female, HCMC 9 male, 9 female; Focus groups MC groups 42% female, HCMC groups 46% female	Interviews: MC 31 White, 1 African-American, HCMC: 2 White, 13 African-American, 2 Native American, 1 Mixed (African/Native American); Focus groups: MC 0% non-white, HCMC 69% non-white	Interviews: Considered at individual level (education and employment) and small area level (additional 18 participants recruited from hospital providing care for many low-income and vulnerable persons); Focus groups: not specified	Interviews: MC 1 to 16, median number of self-reported health conditions 5 (no IQR specified) HCMC 3 to 8, median 5 (no IQR specified) Focus groups 1 to 6, median 3 (no IQR specified)
Favarato	2021	Brazil	Semi-structured interviews	43	Not defined	57.5 (no SD specified)	20 men, 23 women	Not specified	Considered at individual level (working status and schooling)	Mean Elixhauser comorbidity score 4.6 + 1.5
Francis	2020	New Zealand	Qualitative multiple case study research design (two interviews, four weekly contacts with patients over an 18-month period and an interview with their primary health care clinicians)	16	26 to 88	Not specified	7 male, 9 female	6 Māori, 2 Pasifika, 8 European descent	Considered at area level (‘reflective of the population who experience LTCs’)	2 to 7 (calculated from table, no mean or SD specified)
Gill	2014	Canada	Semi-structured interviews with patients, their informal caregivers and family physicians	27 (patients)	67 to 96 (calculated from table)	82.3 (SD 7.7)	16 male, 12 female	Not specified	Considered at individual level (education and accommodation)	1 to 12 (calculated from table), median 5 (no IQR specified)
Hardman	2021	Australia	Semi-structured interviews, 9 face-to-face, 2 by phone (2 interviews were with couples who were both living with MLTCs)	13	47 to 72	61 (no SD specified)	7 male, 6 female (calculated from table)	Not specified	Considered at area level (low income population)	3 to 10, mean 7 (no SD specified)
Heid	2020	USA	In-depth, semi-structured interviews	38	64 to 96	80.05 (SD 9.27)	24 female, 14 male	28 Caucasian, 10 African-American	Considered at individual level (education and income)	2 to 8, mean 4.63 (SD 1.55)
Joensson	2020	Denmark	Narrative (life story) interviews, follow-up formal interviews, informal chats, participant observations	14	66 to 90 (calculated from table)	Not specified	7 men, 7 women	Not specified	Considered at individual level (educational background)	3 to 6 (no mean or SD specified)
Larkin	2021	(*n* = 46 studies) (North America 26,South America 1,Africa 4, Asia 4, Europe 3,Oceania 7, multiple continents 1)	Systematic review (*n* = 46 studies) Interviews 27, focus groups 7, a mix of methods 9, online questionnaires with free text sections 2, ‘conversations’ with participants 1	2631 (*n* = 38 studies)	20 to 90 (*n* = 15 studies)	53.6 (*n* = 28 studies, no SD specified)	(*n* = 41 studies) 1386 (63.3%) male, 799 (36.5%), female, 1 (0.0004%) transgender female, 2 (0.001%) ‘other’	Not specified	Not clearly described for the range of papers (systematic review)	Mean number of conditions 4 (*n* = 20 studies, no SD specified)
Löffler	2012	Germany	Narrative in-depth interviews	19	65 to 85	75 (no SD specified)	6 male, 13 female	Not specified	Considered at individual level (education, vocational training, active at the labour market during lifetime)	Minimum of 3 chronic conditions
Morgan	2019	Ghana	In-depth interviews	20 (Urban area 12, peri-urban “the interface between the urban outskirts of the capital city and the rural countryside” 5, rural 3)	Not defined (eligibility 35 to 75)	Urban 55.6 (SD 10.0), peri-urban 54.0 (SD 11.1), rural 52.7 (SD 10.0)	20 women	Not specified	Considered at individual level (employment and education) and area level (urban, peri-urban and rural)	Mean 2.3 (urban), 2.8 (peri-urban), 4 (rural) (no SDs specified)
Morris	2011	UK	Semi-structured interviews (initial face-to-face interviews, telephone follow-ups and final face-to-face interviews a year later)	21	36 to 84 (calculated from table)	Not specified	11 male, 10 female	Not specified	Considered at individual level (education, occupation and housing tenure)	2 to 6 (calculated from table, no mean or SD specified)
O'Brien	2014	UK	Individual semi-structured interviews (recruitment involved regular telephone discussions with participants…prior to the interview)	14	44 to 64 (calculated from table)	Not specified	6 men, 8 women	Not specified	Considered at area level (living in areas of high socioeconomic deprivation in Scotland)	Range of problems listed in Table 1, minimum of 2
Ørtenblad	2018	Denmark	In-depth and longitudinal study over 18 months—researchers participated in the everyday activities of the informants, observed patients’ appointments, regular phone calls, 2–3 in-depth interviews	10	38 to 65 (calculated from table)	51 (no SD specified)	5 men, 5 women	Not specified	Considered at individual level (education and occupation)	3 to 7 (calculated from table, no mean or SD specified)
Ploeg	2017	Canada	Face-to-face semi-structured interviews with community-living older adults, family caregivers and healthcare providers	41 (older adults)	65 to 85 +	Not specified	23 men, 18 women	Not specified	Considered at individual level (education and household income)	3 to 13, mean 6.3 (no SD specified)
Ploeg	2019	Canada	In-depth, semi-structured in-person interviews	21	65 to 85 +	76.9 (SD 7.4)	11 male, 10 female	Not specified	Considered at individual level (education and household income)	3 to 13, mean 7.4 (SD 2.7)
Porter	2020	UK	Two in-depth qualitative interviews spaced three to six months apart	15	59 to 84	Not specified	7 male, 8 female	14 White British, 1 White non-British	Considered at individual level (employment status and accommodation status)	4 to 8 (calculated from table, no mean or SD specified)
Richardson	2016	USA	Semi-structured, one-on-one interviews	33	51 to 90	Not specified	31 male, 2 female	Race: 6 Black or African-American, 27 White, Ethnicity: 1 Hispanic origin 32 not Hispanic origin	Considered at individual level (education, household income and relationship status)	3 to 11, mean 6 (no SD specified)
Rijken	2021	Netherlands	Face-to-face focus groups, telephone interviews (focussing on a prioritisation exercise), paper questionnaire	883 (20 focus groups/interviews and 863 survey)	40 to 89 (focus groups/interviews) 22 to 96 (survey)	Focus groups/interviews 68.2 (SD 12.3), Survey 70.5 (SD 11.6)	Focus groups/interviews: 7 men, 13 women Survey: Among those who provided information 326 men (43%) and 440 women (57%)	Not specified	SES not addressed for focus groups/interviews or survey	Focus groups/interviews: many participants reported three or more chronic conditions; Survey: all 863 patients living with three or more chronic conditions
Roberto	2005	USA	20-min structured telephone survey and follow-up, face-to-face semi-structured interviews (approx 2 h)	17	69 to 84	76.1 (SD 5.37)	17 women	Not specified	Considered at individual level (education, monthly income and living situation)	2 to 6 (calculated from table, no mean or SD specified)
Sand	2021	Denmark	Individual semi-structured interviews	9	38 to 65	54.1 (no SD specified)	3 male, 6 female	Not specified	Considered at individual level (education and occupation)	At least 3 (no mean or SD)
Sav	2013	Australia	Semi-structured in-depth interviews (face-to-face or over the phone) with people with chronic illness and their carers	97 total, 85 people with chronic illness (Consumer only 69, Carer only 12, Carer/consumer 16)	16 to 83	57.2 (no SD specified)	32 male, 65 female	23 Aboriginal and Torres Strait Islander (indigenous person) 19 Culturally and linguistically diverse (e.g. Egyptian, Lebanese, Japanese, Burmese, Italian, Samoan) 55 Caucasian	Considered at area level (four regions of Australia purposively selected as they represent considerable socioeconomic, cultural, geographical (e.g. metropolitan or rural/remote) diversity)	65.9% had three or more chronic illnesses (no mean or SD specified)
Sells	2009	USA	Semi-structured interviews—three qualitative interviews at approximately 4-month intervals, this study reports from the first qualitative interview	33	Not defined	50.5 (no SD specified)	10 men, 23 women	21 Caucasian, 12 African- American, 9 Hispanic	Considered at area level (patients at the PCC [Primary Care Center] reflect a largely racial/ethnic minority, low income, urban population)	Not clearly specified, all participants carried multiple medical diagnoses, (no range, mean or SD specified)
Shin	2022	9 studies (Sweden 3, Norway 2, Canada 1,Denmark 1, Netherlands 1, UK 1)	Meta-ethnography (*n* = 9) Semi-structured interviews 6, individual interviews 2, narrative interviews 1	173	68 to 95	Means for each of the 9 studies: 88, 79, 84.4, 80.6, 78, 84, 89, 84, 85.5 (no SDs specified)	63 men, 110 women	Not specified	Not clearly described for the range of papers (systematic review)	Not clear
Slightam	2018	USA	Paper and electronic surveys (mixed methods)	387 (survey responses)	Not defined	62.5 (SD 12)	(n = 381): 316 male, 65 female	Race (*n* = 377) 320 White, 25 Asian, 21 African American, 14 American Indian or Alaska Native, Ethnicity (*n* = 382) 31 Hispanic/Latino, 351 Non-Hispanic/Latino	Considered at individual level (education, annual household income and employment)	2 to 10 or more, mean 4.3 (SD 2.1)
Sun	2022	China	Semi-structured in-depth interviews with couples	16 (couples)	62 to 86 (for patients, calculated from table)	73.3 (SD 7.61)	16 couples: 10 male patients, 6 female patients	Not specified	Considered at individual level (education level and family monthly income)	2 to 5 (for couples)
Townsend	2008	UK	Two in-depth, semi-structured interviews, symptom diary	23	‘aged about 50 years'	Not specified	10 men, 13 women	Not specified	Considered at individual level (employment) and area level (people from a range of socioeconomic backgrounds)	4 or more
van Merode	2018	Netherlands and Belgium	Individual, semi-structured interviews	22	45 to 91	Men 72, women 70 (no SDs specified)	7 men, 15 women	Not specified	Considered at individual level (patients with a variety of characteristics were invited to ensure…diversity of socioeconomic status)	2 to 5 (calculated from table, no mean or SD specified)
White	2016	Australia	Two interviews (not formally defined, lasted 60–120 min), field notes	16	20 to 67 (calculated from table)	Not specified	5 male, 11 female (calculated from table)	Not specified	Considered at individual level (education, occupation and social/living situation)	1 to 5 (calculated from table, more than 50% of participants with 3 or more, no mean or SD specified)
Zulman	2015	USA	Screening survey and focus groups	53 (likely subset of Slightam)	Not defined	59 (SD 11)	39 male, 14 female	Race (*n* = 52) 43 White, non-Hispanic, 3 Black, non-Hispanic, 5 Hispanic, 7 Other, non-Hispanic (Individuals could indicate more than one response item)	Considered at individual level (employment, education and annual household income)	3 to 7 or more, mean 5 (SD 2)

*SD* standard deviation, *IQR* inter-quartile range, *SES* socioeconomic status

Of the 46 included studies, 42 were primary research studies and four were qualitative syntheses [61, 66, 75, 89]. The four qualitative syntheses included a total of 19 of the 46 primary research studies also included in our review [25, 52, 54–56, 59, 60, 64, 65, 67, 71, 76, 78–81, 87, 88, 92]. There were additionally four duplicate populations within the 42 included primary research studies [59, 60, 64, 65, 67, 68, 90, 94].

The number of participants ranged from nine to 883 in the primary research studies, and from 173 to 2631 in the systematic reviews. The total number of participants with MLTCs across all 46 studies was over 5000 (the exact total number is difficult to ascertain due to duplicate populations and lack of clarity within some studies).

Of the 42 primary research studies, 36 used interviews as their data collection method, one used focus groups, two used both focus groups and interviews and one used surveys (mixed methods). One study was a multiple case study over 18 months and one study used triangulation of data from electronic health records, observation of primary care consultations and interviews. The four systematic reviews contained studies using a range of data collection methods, with interviews being the most common.

19 papers reported numbers of men/women (including one systematic review), 24 papers reported numbers of males/females, one systematic review reported numbers for males/females/transgender females/’other’ for a subset (41) of 46 studies and two systematic reviews did not report numbers (one reported the majority of studies recruited predominately more women).

Thirty-one studies did not clearly report the ethnicity and/or race of participants. Two studies reported 100% white participants, five studies recruited mostly white participants, three studies recruited mostly Caucasian participants and one study recruited Chinese participants. One study reported a minority of black participants only. One study had four cohorts—two cohorts recruited predominantly white participants, one cohort recruited predominantly non-white participants and one cohort recruited predominantly African-American participants. One study had representation from individuals of Māori, Pasifika and European descent, and in one systematic review most of the included studies did not report ethnicity or the majority of participants were white.

Comparison across studies for socioeconomic status was difficult due to inconsistent reporting. 29 papers considered individual level factors such as income, health insurance provider, education level, employment level and living situation. Six papers considered socioeconomic status at area level and three papers considered both individual and area level. Eight papers did not cover socioeconomic status, or it was poorly described. Six papers specifically noted socioeconomic diversity.

Study locations for primary research studies were: 11 USA, six UK, seven Canada, four Denmark, three Sweden, three Australia, two China, one New Zealand, one Netherlands, one Netherlands and Belgium, one Ghana, one Germany, one Brazil. The four systematic reviews included studies across several countries. Most studies included a wide age range of participants, though 14 studies focused on older age groups (65 +).

### Quality appraisal

**Fig. 2:**
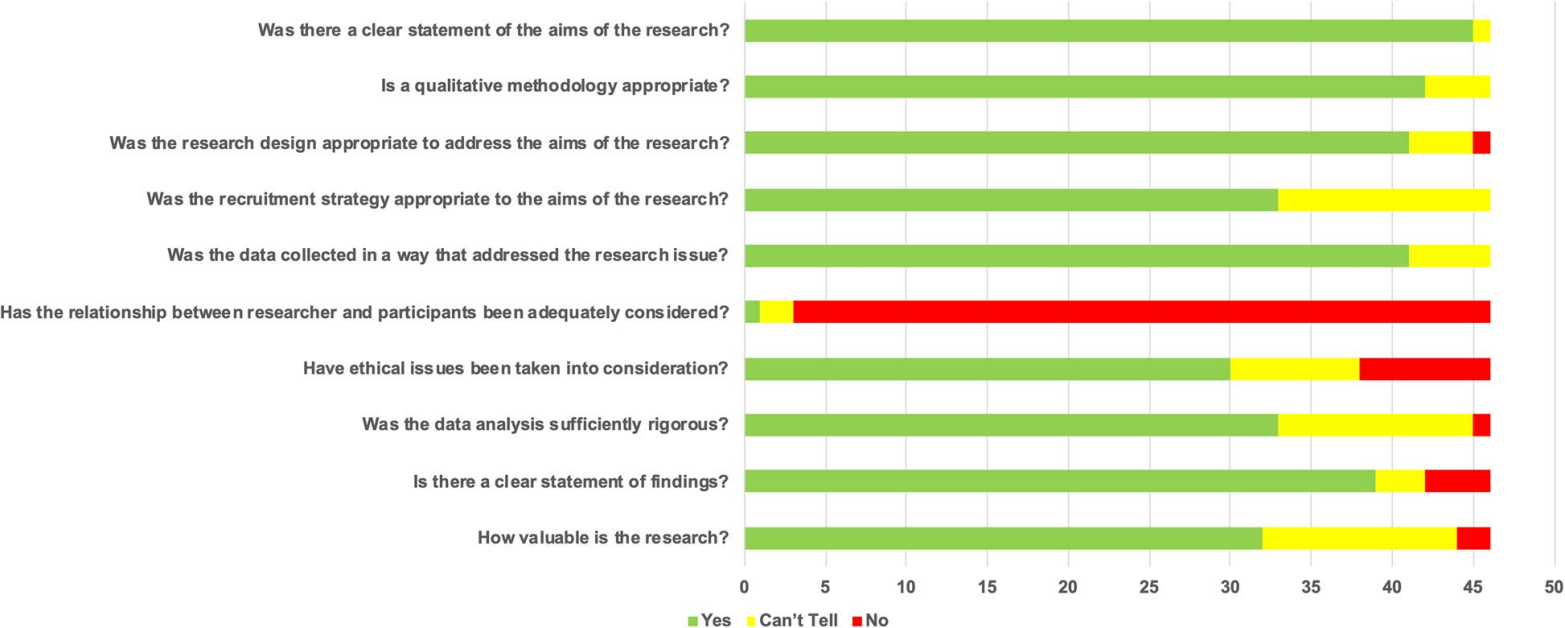
Summary of the quality assessment of included studies

The overall quality of the included papers was generally high, although researcher reflexivity, discussion around researchers’ responses to situations occurring during the research, and acknowledgement of how any changes in the protocol impacted on the research was under-reported in many studies (Fig. 2).

### Thematic synthesis

**Fig. 3:**
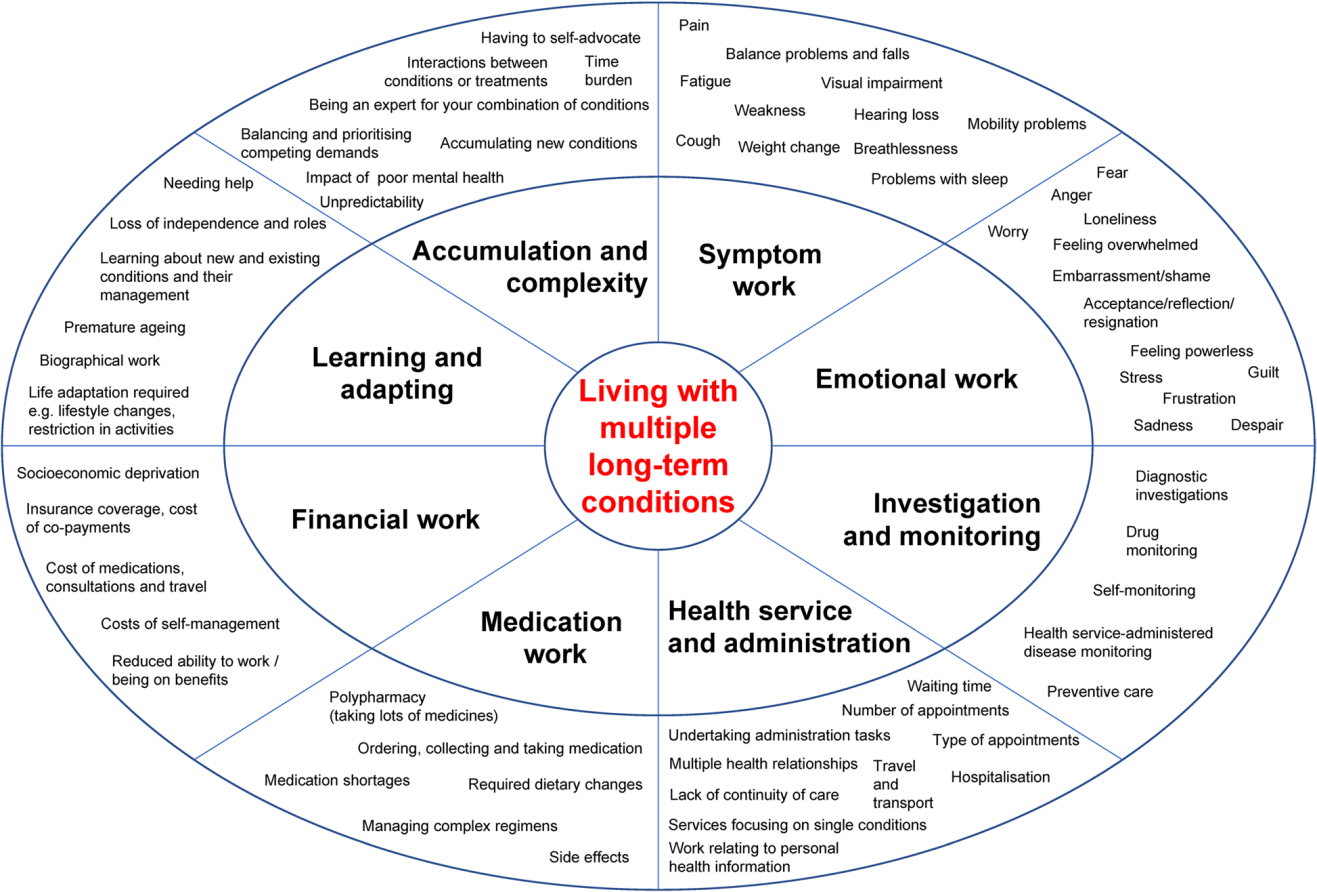
Themes of work. Footnote: The outer oval includes examples of concepts in each theme (not an exhaustive list)

Eight overarching themes were generated to represent the work of living with MLTCs. These were accumulation and complexity, learning and adapting, investigation and monitoring, medication work, health service and administration, symptom work, emotional work and financial work. Further detail is given in Fig. 3 and Table 3.

**Table 3:** Themes, concepts and quotes from included studies

Theme	Description	Example concepts	Example quotes (participant quotes italicised, author quotes non-italicised)
Accumulation and complexity	The additional burden for individuals who are living with multiple, rather than just one, long-term condition	Complex management Constant decisions and prioritisations Multiple interactions (diseases/treatments) Unpredictability (varying impact) Daily burden Endless, lifelong work Self-reliance and ownership (being the person who understands your conditions best, deciding what is important and what to disclose to health professionals) Multiple health relationships New and additional illnesses Not getting a diagnosis Living with uncertainty Importance of individual context Burden and exhaustion due to self-care Balancing work, appointments and treatment Impact of poor mental health Time burden	Aberg et al “The complexity of living with several simultaneous health problems is that its intensity and impact on daily life can vary from time to time, from day to day but also during the day” Zulman et al “patients with MCCs [multiple chronic conditions] often feel that they must serve as their own expert and advocate for their needs” Francis et al “Although the self-management approach may be entirely appropriate for people with a single, early stage LTC, there is little congruence between the self-management approach’s idealised expert patient and this study’s exhausted participants” Duguay et al “The multiple medications (polypharmacy) required for the treatment of multiple chronic diseases cause other problems for most participants, adding to the complexity of the situation. For example, some drugs have side effects that require treatment with other drugs, which contributes to increasing polypharmacy” Ørtenblad et al “Several of the informants experienced conflicts between managing their diseases and treatments and their work life. Christine said that all although she is fairly young, she is never asked how appointments fit with her work schedule, perhaps, she wonders, because people suffering from multiple diseases are not expected to have jobs”
Learning and adapting	Learning about new and existing conditions and their management; physical and psychological adaptations required to live with MLTCs	Self-management, including required lifestyle modification e.g. diet, physical activity Biographical work including premature ageing, comparing self with others Restriction in activities – activities of daily living, social activities etc Dependency on others Loss of autonomy Interpersonal challenges Social losses Impact on ability to work Limitations on travel e.g. cannot drive car, impacted by medications Assessment work Trying to understand the cause of problem and how to manage symptoms Regular reappraisal of conditions Conflicting goals between patients and healthcare professionals (and lack of discussion) Seeking information Impacting on family and social networks Learning about conditions and care (acquiring new health knowledge/developing health literacy)	Clarke and Bennett (2013) [60] “Specifically, she had made changes to her routines, educated herself about her various illnesses, begun to take four prescribed medications, employed various non- prescription drugs, and tried a number of home remedies” Duguay, Gallagher and Fortin “Regardless of the participant’s age, in their eyes, chronic diseases and the numerous associated physical and social limitations evoke old age” Aberg et al “On one hand, a sense of loss of how life once was is described. The health problems limit the possibilities for what previously made sense in life such as walking, going to the movies, restaurants and concerts, accessing driving vehicles, visiting the grandchildren and the cemetery. *“I would like to bake bread. I always did that and cookies … what fun it was. I can’t stand up for that long because of my back. So, no point in thinking about it*””
Investigation and monitoring	Specific investigations and monitoring work associated with MLTCs	Multiple diagnostic tests Blood pressure monitoring Blood sugar monitoring Blood tests Monitoring insulin dosage Attending regular appointments Monitoring of new medications Preventive care (can be more complicated for people living with MLTCs)	Gill et al “Patients also noted poor coordination among providers when multiple medications had to be prescribed and various tests and procedures had to be coordinated. One patient attempted to schedule two imaging appointments from two different providers, but was hindered by the system’s inability to coordinate the scans” Ancker et al “One man explained why he had not followed up on a potential route to get insurance coverage for the shingles vaccine. *“Who wants to go through all that? Who has the time and energy to continue the struggle, especially someone who is chronically trying to deal with everything else they’ve got to deal with? … It’s hard enough when you’re healthy and you’re with it, and you’re feeling good… When you’re not feeling well at all, it’s difficult. I don’t have the energy. I don’t have the time. I don’t feel good. I don’t want to deal with it.*”” Bardach et al “*For the last 3 years I have went through just about every test that they have got to find out what was wrong with me, and I just worn down until I am tired. I’m tired of going to the doctor. I’m tired of doing what the doctor says … I just want to rest and then I will go and have some more* [preventive] *things done later*” Slightam et al “*I am tired of feeling like a pin cushion. I am tired of the swings in my blood sugars*”
Medication work	The work associated with taking and managing medications	Polypharmacy Managing complex regimens Side effects, adverse effects and impairing health Medication adherence Medication interactions	Zulman et al “*It’s a struggle. It is! It’s a huge struggle. Every week I have to put my meds in pill boxes because if I don’t do that, with as many different medicines as I’m taking…To be perfectly honest I couldn’t even tell you how many pill bottles it really is.*” Sand et al “*I know that the day after* [she had injected the weekly medication]*, I’m not capable of doing much. But I do take my medicine, because I respect authorities, and I feel that it helps. But these are the trade-offs to me; on one hand, to feel that it’s helping me, but on the other hand, I have to live with the side effects. That’s like choosing between plague and cholera.* (Female, 46 years)” Van Merode et al “Having to take multiple medications is a major aspect of the treatment burden. Interactions, side effects, and change of brands because of government policy added greatly to the burden patients experienced” Sav et al.: “Some participants described the frustration they experienced about the inconvenience of having to rely on medication, especially when it interfered with daily activities, such as shopping and employment. Many participants also felt uncomfortable about their treatment, a finding that appeared to be related to the stigma associated with medication use and chronic illness. Male participants, particularly those from a CALD [culturally and linguistically diverse] background, commented about how the use of medication reminded them of their illness. They often seemed troubled by the idea of having to rely on medication for the rest of their lives”
Health service and administration	Work specifically related to health services	Multiple appointments Type of appointments Multiple health relationships Communication and relationship issues Conflicting goals between patients and healthcare professionals (and lack of discussion) Travel and transport Fragmentation of care/services focus on single conditions Continuity of care Access issues and waiting times Hospitalisation Work relating to personal health information e.g. keeping health professionals up to date, monitoring health record and blood results, dealing with errors, keeping a list of medication for when it is needed, etc.) Non-attendance due to cost/difficulty	Ploeg et al. (2017) “Older persons with MCC and caregivers described challenges receiving services from multiple providers who focus on a single disease or single aspect of their health, and do not see them as a whole person. Care is often experienced as disjointed and lacking coordination” Morgan et al “On the one hand individual disease management promotes efficiency and continuity of care, for example through the scheduling of subsequent follow-up appointments; on the other hand it may result in the neglect of other co-morbidities and these not being discussed at the clinic” Ancker et al “the work they performed to manage records or correct their information was generally invisible to their health care providers. This invisibility raised new challenges: patients found these tasks interfering with their regular illness work and felt they had nowhere to turn for assistance. *“Nobody wants to help you,”* said one” Ancker et al.: “One of the biggest issues facing patients is the enormous amount of difficult, frustrating, and emotionally tiring work involved in addressing informational errors. Because this work is conducted outside of the relationship with any individual health care provider, it is often invisible to their health care providers. Furthermore, because this invisible work arises from complexities in medical care and medical coverage, it seems likely to fall most heavily on those with the most encounters with the medical system, constituting a systemically regressive tax on illness” Eckerblad et al. (2015) “All were dependent on support in one way or another from health care. Different diseases resulted in visits to several different health care departments for checkups. Even though they knew they ought to go and felt guilty about not doing so, the effort to plan transportation and wait their turn in line just took too much energy”
Symptom work	The wide range of symptoms experienced by people living with MLTCs	Pain Physical limitations Fatigue, exhaustion, tiredness and lack of energy Problems with sleep Low mood Problems with eyesight and hearing Respiratory/breathing problems Balance problems/falls Weight change – weight gain or weight loss Decreased physical strength Cough Reduced cognitive ability Nausea Breathlessness	Coventry et al “This was highlighted by data that illustrated the way in which multimorbidity had imposed severe restrictions on mobility, more so than with the effects of single conditions” Duguay et al “Not all diseases have the same significance in the overall multimorbidity experience. Those that involve pain are more distressing than those whose symptoms are more difficult to detect” Clarke and Bennett (2013) [60] “*so I haven’t seen my doctor for over a year. Mind you I don’t really want to. There’s nothing that she can do. My spine is extremely painful but there’s nothing they can do with that…*” Eckerblad et al. (2020) “For some participants, the symptoms were so severe and debilitating that they had no alternative but to passively try to endure. They described it as a vegetative life, and they tried to cope from one day to the next”
Emotional work	The multiple emotions experienced by patients as a result of living with MLTCs	Worry Frustration Embarrassment Acceptance and reflection Resignation Recognition of how MLTCs impact on others (for example guilt and feelings of being a burden) Hope Loneliness and isolation Overwhelmed and worn out Sadness, crying Powerlessness Anger, disgust Despair Fear Feelings of loss Shame (for example due to reduced ability to work, change in appearance) Stress Desire for independence	O’Brien et al “She described how she struggled to make sense of the changes that her many illnesses had brought. When she was able to be active around the home, she found it helped distract her from feelings of anger (that her life so little resembled that of her peers), grief (that her illness had resulted in so many losses, particularly paid employment which had been an important part of her identity), and fear (about her health and how it would impact everyday life work in future)” Sand et al *“What bothers me the most is that I don’t want to be a burden to others. I don’t want to be a hassle to anyone. No one should have to take my needs into consideration. That is hard to accept.”* Eckerblad et al. (2015) “Feeling worried, nervous or sad made it hard to sleep, and some reported problems of being so emotional and easily moved that they could suddenly start crying in an uncontrolled manner” Clarke and Bennett (2008) “For example, a 77-year-old man who had arthritis, back problems, diabetes, heart disease, kidney disease and a thyroid condition expressed his resignation in this way: *‘That’s life…I’m not happy with it but if it has to be that way, it has to be. That’s all there is to it. You just accept it and move on.*’” Shin et al “most participants in this review experienced psychological instability related to feelings of guilt regarding receiving care from their families to self-perceptions of being a burden to their informal caregivers and a sense of meaninglessness”
Financial work	The financial impact of living with MLTCs	Cost of medication and consultations Transport costs and parking costs Balancing healthcare costs with other expenditure – impact on other aspects of daily life Private healthcare, out of pocket payments, reimbursement issues etc Dependency on family Socioeconomic deprivation shapes the experience of multimorbidity Medication Cost of self-management (e.g. diet, gym membership, chiropractor) Reduced ability to work Benefits being insufficient	Hardman et al “Increased healthcare costs were often complicated by loss of income. As multimorbidity increased, functional capacity declined, with ten of the thirteen participants reporting that their health conditions had forced them to stop work” Larkin et al “Participants also discussed losing their savings, losing their home and accruing high levels of debt in order to meet the high costs associated with multimorbidity.” Larkin et al “*All my money goes on my health aside from basic bills. I do not buy treats, clothes, haircuts, toiletries, things for the house*” Morgan et al “For women in the Greater Accra Region, there was an evident reliance on the health care system, although this was met by inconsistent coverage under the NHIS. Despite the availability of a health insurance package, the majority of women experienced a financial burden related to meeting their health care demands, and were dependent on family and community members to offset this” O’Brien et al.: “Participants’ accounts implicitly reveal the effects of deprivation (which included descriptions of having to manage many problems and having access to few social and material resources), and how these were perceived to exacerbate their struggles to manage, especially when combined with a mental health problem” Bardach et al *“When you are taking 20 bottles of medicine, and you have anywhere from 24 to 30 dollars to pay, on top of all them doctors you just had to pay for, it’s hard. If you go to one doctor to the next, they will change everything you are on, even though you are doing fine on the medications that you are on because they want you on their medications*”

The eight themes were often overlapping, reflecting the complex and holistic reality of the lived experience of MLTCs. Some concepts aligned with more than one theme, for example the practical work of polypharmacy naturally fit into medication work but we argue that drug interactions and the additional work when a new medication is added equally corresponds with our accumulation and complexity theme. Time burden was an important factor across many themes. For example, papers described people with MLTCs having to invest considerable time and effort undertaking investigations and monitoring and self-management tasks, attending appointments and organising medications. The impact of poor mental health was also identified as having wide-ranging influence on many areas such as self-management, organising healthcare, adherence to medications and social activities. In view of the way both added to the complexity of living with MLTCs, time burden and the impact of poor mental health were included in the accumulation and complexity theme.

#### Accumulation and complexity

Here, the additional work for individuals who are living with multiple, rather than just one, LTC is described. Although the nature of individual conditions is important, we identified common difficulties experienced by many people living with MLTCs. These included accumulating new and additional conditions over time, not receiving a diagnosis, interactions between diseases, symptoms or treatments, the need to make constant decisions and prioritisations, and unpredictability/uncertainty. An example was described by Aberg and colleagues [52]:

“The complexity of living with several simultaneous health problems is that its intensity and impact on daily life can vary from time to time, from day to day but also during the day”.

Balancing work, appointments and treatment was challenging for participants, as noted in Ørtenblad and colleagues [79]:

“Several of the informants experienced conflicts between managing their diseases and treatments and their work life…although she is fairly young, she is never asked how appointments fit with her work schedule, perhaps, she wonders, because people suffering from multiple diseases are not expected to have jobs”

Living with MLTCs involves complex management associated with the coordination of multiple health relationships, above and beyond those for people with an individual LTC. Given the great variety in the nature of conditions, their combinations and challenges, patients commonly understand their individual circumstances better than others, and further work results from deciding what is important and what to disclose to health professionals. This self-reliance and ownership work was described by Zulman and colleagues [94]:

“…patients with MCCs [multiple chronic conditions] often feel that they must serve as their own expert and advocate for their needs”.

Other concepts within this theme were daily burden and endless, lifelong work. Burden and exhaustion due to self-care was specifically noted. The many different, and sometimes conflicting, self-management tasks for various conditions can require a large degree of effort, and sometimes be overwhelming, even leading to healthcare disengagement, as described by Francis and colleagues’ [70]:

“Although the self-management approach may be entirely appropriate for people with a single, early stage LTC, there is little congruence between the self-management approach’s idealised expert patient and this study’s exhausted participants”.

Finally, this theme highlighted the importance of the individual context of MLTCs. MLTCs are experienced by participants within a context determined by issues such as urban or rural environment, housing, employment and financial circumstances, together with the individual’s previous life events and education, social circumstances and support structures.

#### Learning and adapting

This theme encompasses the work that is required to learn about new and existing conditions and their management, and necessary life adaptation and lifestyle changes. Participants sought a variety of health information, particularly around LTCs, medications and interactions, and how to improve self-care skills.

Participants undertook regular assessment work, for example reappraisal of their conditions, comparing themselves with others and trying to understand the cause of problems and how to manage symptoms. There were sometimes conflicting goals between patients and healthcare professionals (and lack of discussion).

Self-management was frequently discussed. Common self-management activities were dietary and physical activity changes, but there were many other examples such as breathing exercises, stretching, applying heat/ice, acupuncture and massage along with lifestyle modifications such as reducing work hours and resting. An example was described in a study by Clarke and Bennett [60]:

“Specifically, she had made changes to her routines, educated herself about her various illnesses, begun to take four prescribed medications, employed various non-prescription drugs, and tried a number of home remedies”.

The learning and adapting theme also encompasses biographical work, the disruption to people’s identity and sense of self, which can prompt a grief reaction. There was clear recognition of social losses including increasing isolation, a restriction in social activities, feelings of premature ageing and loss of independence and roles. Many found it challenging to adjust to restrictions as described by Duguay, Gallagher and Fortin [63]:

“Regardless of the participant’s age, in their eyes, chronic diseases and the numerous associated physical and social limitations evoke old age”.

Finally, increasing loss of autonomy and dependency on others were important concepts. Limitations on travel may be caused by no longer being able to drive a car or due to complex medication regimens or side effects. A restriction in the ability to carry out activities of daily living often led to needing help from others. This was linked to interpersonal challenges, for example the impact of MLTCs on family and social networks, both on the nature of the relationship itself and on practical impacts, particularly for the spouse or closest relative. An example was described by Aberg and colleagues [52]:

“On one hand, a sense of loss of how life once was is described. The health problems limit the possibilities for what previously made sense in life such as walking, going to the movies, restaurants and concerts, accessing driving vehicles, visiting the grandchildren and the cemetery. *“I would like to bake bread. I always did that and cookies … what fun it was. I can’t stand up for that long because of my back. So, no point in thinking about it”*”

#### Investigation and monitoring

Most LTCs require an element of investigation and monitoring, although this is dependent on the condition, for example the monitoring work associated with diabetes is very high. This theme encompasses both self-monitoring, for example patient monitoring of blood sugars, blood pressure readings, insulin dosages, blood test values and side effects from new medications, and predominantly health service-administered disease monitoring (with the need to sometimes attend regular appointments), for example blood tests, multiple diagnostic tests/investigations and the monitoring of specialised medication.

This was illustrated by Gill and colleagues [71]:

“Patients also noted poor coordination among providers when multiple medications had to be prescribed and various tests and procedures had to be coordinated. One patient attempted to schedule two imaging appointments from two different providers, but was hindered by the system’s inability to coordinate the scans”

Notably, participation in preventive care such as screening can be more challenging for those with MLTCs due to issues such as current poor health, mobility issues and insufficient time and energy. This can lead to non-engagement as demonstrated in Ancker and colleagues [53]:

“One man explained why he had not followed up on a potential route to get insurance coverage for the shingles vaccine. *“Who wants to go through all that? Who has the time and energy to continue the struggle, especially someone who is chronically trying to deal with everything else they’ve got to deal with? … It’s hard enough when you’re healthy and you’re with it, and you’re feeling good… When you’re not feeling well at all, it’s difficult. I don’t have the energy. I don’t have the time. I don’t feel good. I don’t want to deal with it.”*”

#### Medication work

Polypharmacy is a major source of work (and treatment burden) for patients living with LTCs. The high number of medications taken per day results in complex and possibly confusing regimens, with potentially high cost and a significant time burden for ordering, collecting and taking medication.

This experience was described by Zulman and colleagues [94]:

“*It’s a struggle. It is! It’s a huge struggle. Every week I have to put my meds in pill boxes because if I don’t do that, with as many different medicines as I’m taking…To be perfectly honest I couldn’t even tell you how many pill bottles it really is.”*

People living with MLTCs also have to cope with significant side effects and drug interactions (both with other conditions and other medications), as demonstrated by both Sand and colleagues and Van Merode and colleagues’ [29, 86]:

“*I know that the day after* [she had injected the weekly medication]*, I’m not capable of doing much. But I do take my medicine, because I respect authorities, and I feel that it helps. But these are the trade-offs to me; on one hand, to feel that it’s helping me, but on the other hand, I have to live with the side effects. That’s like choosing between plague and cholera.* (Female, 46 years)”

“Having to take multiple medications is a major aspect of the treatment burden. Interactions, side effects, and change of brands because of government policy added greatly to the burden patients experienced”.

Polypharmacy can have a detrimental impact on the ability to attend activities, make plans and travel. Medication adherence can be affected by complex regimens, but also by fear of side effects, drugs causing harm, stigma, required dietary changes and medication shortages. These concepts are discussed by Sav and colleagues’ [87]:

“Some participants described the frustration they experienced about the inconvenience of having to rely on medication, especially when it interfered with daily activities, such as shopping and employment. Many participants also felt uncomfortable about their treatment, a finding that appeared to be related to the stigma associated with medication use and chronic illness. Male participants, particularly those from a CALD [Culturally and linguistically diverse] background, commented about how the use of medication reminded them of their illness. They often seemed troubled by the idea of having to rely on medication for the rest of their lives”

#### Health service and administration

This theme relates to the work specifically concerning health services (part of treatment burden), most notably the high number of appointments required for people living with MLTCs, often with different doctors and in different departments and/or healthcare facilities. There is a clear negative impact for patients with MLTCs due to fragmentation of care with services focusing on single diseases, as illustrated by Ploeg and colleagues [80]:

“Older persons with MCC and caregivers described challenges receiving services from multiple providers who focus on a single disease or single aspect of their health, and do not see them as a whole person. Care is often experienced as disjointed and lacking coordination”.

This phenomenon was also noted in Morgan and colleagues [76]:

“On the one hand individual disease management promotes efficiency and continuity of care, for example through the scheduling of subsequent follow-up appointments; on the other hand it may result in the neglect of other co-morbidities and these not being discussed at the clinic”.

Multiple health relationships and a lack of continuity of care both lead to a substantial amount of ‘invisible’ work carried out by patients relating to managing personal health information, for example the transfer of information between providers, keeping a list of medication for when it is needed and managing errors [53]. Ancker and colleagues’ study describes [53]:

“the work they performed to manage records or correct their information was generally invisible to their health care providers. This invisibility raised new challenges: patients found these tasks interfering with their regular illness work and felt they had nowhere to turn for assistance. *“Nobody wants to help you,”* said one”.

“One of the biggest issues facing patients is the enormous amount of difficult, frustrating, and emotionally tiring work involved in addressing informational errors. Because this work is conducted outside of the relationship with any individual health care provider, it is often invisible to their health care providers. Furthermore, because this invisible work arises from complexities in medical care and medical coverage, it seems likely to fall most heavily on those with the most encounters with the medical system, constituting a systemically regressive tax on illness”.

Along with communication and relationship issues with healthcare providers, other sources of work were practical issues related to travel and transport. These included time burden and cost, the required planning and an acknowledgement of the increased impact on those in rural areas. Access issues and waiting times were also described, together with the impact of hospitalisation with the link to changes in medication and the impact on others. For some, the cost and difficulty associated with appointments led to non-attendance, linking to the work demonstrating an association between people who miss appointments and high treatment burden and was illustrated by Eckerblad and colleagues [64, 95]:

“All were dependent on support in one way or another from health care. Different diseases resulted in visits to several different health care departments for checkups. Even though they knew they ought to go and felt guilty about not doing so, the effort to plan transportation and wait their turn in line just took too much energy”.

#### Symptom work

This theme relates to the wide range of symptoms experienced by people living with MLTCs. Key symptoms emerging from this evidence synthesis were pain, physical limitations/mobility problems and fatigue/exhaustion/tiredness/lack of energy, as illustrated by the two following extracts from Coventry and colleagues and Duguay and colleagues [61, 63]:

“This was highlighted by data that illustrated the way in which multimorbidity had imposed severe restrictions on mobility, more so than with the effects of single conditions”

“Not all diseases have the same significance in the overall multimorbidity experience. Those that involve pain are more distressing than those whose symptoms are more difficult to detect”

In total, over one hundred individual symptoms were expressed by participants of the studies.

Other symptoms included problems with sleep, low mood, problems with eyesight and hearing, respiratory/breathing problems, balance problems/falls, weight change (gain or loss), decreased physical strength, cough, reduced cognitive ability and nausea. Patients did not always seek medical help for their symptoms as the following extract from Clarke and Bennett illustrates [60]:

“*so I haven’t seen my doctor for over a year. Mind you I don’t really want to. There’s nothing that she can do. My spine is extremely painful but there’s nothing they can do with that…”*

#### Emotional work

This large theme encompasses the significant emotional impact of living with MLTCs, both due to the direct impact of MLTCs on individuals and additionally due to the recognition of how MLTCs impact on others as described by both Sand and colleagues and Shin and colleagues [86, 89]:

*“What bothers me the most is that I don’t want to be a burden to others. I don’t want to be a hassle to anyone. No one should have to take my needs into consideration. That is hard to accept.”*

“most participants in this review experienced psychological instability related to feelings of guilt regarding receiving care from their families to self-perceptions of being a burden to their informal caregivers and a sense of meaninglessness”.

Commonly described concepts included worry, frustration, guilt, loneliness and feeling isolated, sadness, feeling overwhelmed, anger, despair, embarrassment/shame/disgust (for example due to reduced ability to work, change in appearance), fear, stress and feeling powerless. Eckerblad and colleagues describe this emotional work [64]:

“Feeling worried, nervous or sad made it hard to sleep, and some reported problems of being so emotional and easily moved that they could suddenly start crying in an uncontrolled manner”.

Many individuals also described a desire for independence and feelings of loss, as described by O’Brien and colleagues [78]:

“She described how she struggled to make sense of the changes that her many illnesses had brought. When she was able to be active around the home, she found it helped distract her from feelings of anger (that her life so little resembled that of her peers), grief (that her illness had resulted in so many losses, particularly paid employment which had been an important part of her identity), and fear (about her health and how it would impact everyday life work in future)”.

Other participants described more positive emotions such as hope and acceptance as described in Clarke and Bennett [58]:

“For example, a 77-year-old man who had arthritis, back problems, diabetes, heart disease, kidney disease and a thyroid condition expressed his resignation in this way: *‘That’s life .. . I’m not happy with it but if it has to be that way, it has to be. That’s all there is to it. You just accept it and move on.’*”

#### Financial work

This theme details the impact that MLTCs has on finances. MLTCs leads to a reduced ability to work, thus reducing income and limiting opportunities. Additionally, the cost of medications, consultations, self-management (for example diet, gym membership, chiropractor) and travel (transport and parking costs) are a substantial financial burden for many. Having MLTCs resulted in more consultations and often more medication, thus those with MLTCs had increased healthcare costs, as described by Hardman and colleagues [72]:

“Increased healthcare costs were often complicated by loss of income. As multimorbidity increased, functional capacity declined, with ten of the thirteen participants reporting that their health conditions had forced them to stop work”.

In several countries, insurance coverage, out of pocket payments/copayments and the administration required for insurance and reimbursement led to further work for patients. Medications not taken, delayed treatment, and consultations not attended all occur as a direct consequence of cost. The financial consequences of MLTCs also led to a need to balance healthcare costs with other expenditure, and the significant impact of this on many other aspects of daily life, for example the ability to pay bills, afford family and leisure activities, and implications on savings, debt and housing, as illustrated by Larkin and colleagues [75]:

“Participants also discussed losing their savings, losing their home and accruing high levels of debt in order to meet the high costs associated with multimorbidity.”

“*All my money goes on my health aside from basic bills. I do not buy treats, clothes, haircuts, toiletries, things for the house*”

Socioeconomic deprivation shapes the experience of multimorbidity. For example, participants described how benefits were insufficient for financial security with some being dependent on family members. Morgan and colleagues describe [76]:

“For women in the Greater Accra Region, there was an evident reliance on the health care system, although this was met by inconsistent coverage under the NHIS. Despite the availability of a health insurance package, the majority of women experienced a financial burden related to meeting their health care demands and were dependent on family and community members to offset this”.

Finally, a negative social environment can directly impact on the ability of people to cope with MLTC, as described by O’Brien and colleagues [78]:

“Participants’ accounts implicitly reveal the effects of deprivation (which included descriptions of having to manage many problems and having access to few social and material resources), and how these were perceived to exacerbate their struggles to manage, especially when combined with a mental health problem”.

### Confidence in findings

For our GRADE-CERQual summarised review finding ‘People living with MLTCs do not just experience one type of work, but multiple, and these occur in differing combinations depending on the nature and combination of conditions and other factors’ we identified ‘No/Very minor concerns’ for methodological limitations, coherence, adequacy, and relevance leading to ‘High confidence’ in our finding.

We had ‘High confidence’ in all other findings except ‘Biographical work—the impact of MLTCs on self-perception and life narrative’ and ‘The impact of having MLTCs on time, including the time lost to healthcare activities, lacking time for medical interaction, time spent on administrative activities, time undertaking self-care, balancing with other activities such as work’ (both ‘Moderate confidence’ with ‘Minor concerns’ for adequacy because these themes were not present in all studies). We additionally had ‘Minor concerns’ regarding relevance for ‘Financial work as a theme of burden’ because of the variation in study populations, geographical locations and health and social care systems represented in the different studies, and for ‘Learning and adapting work as a theme of burden’ because the need to adapt and learn varied by factors such as the specific long-term conditions being considered and the differing characteristics of the study participants (e.g. age, gender, socioeconomic status). Further details are provided in Supplementary Table 3.

### Coverage of themes by included papers

**Fig. 4:**
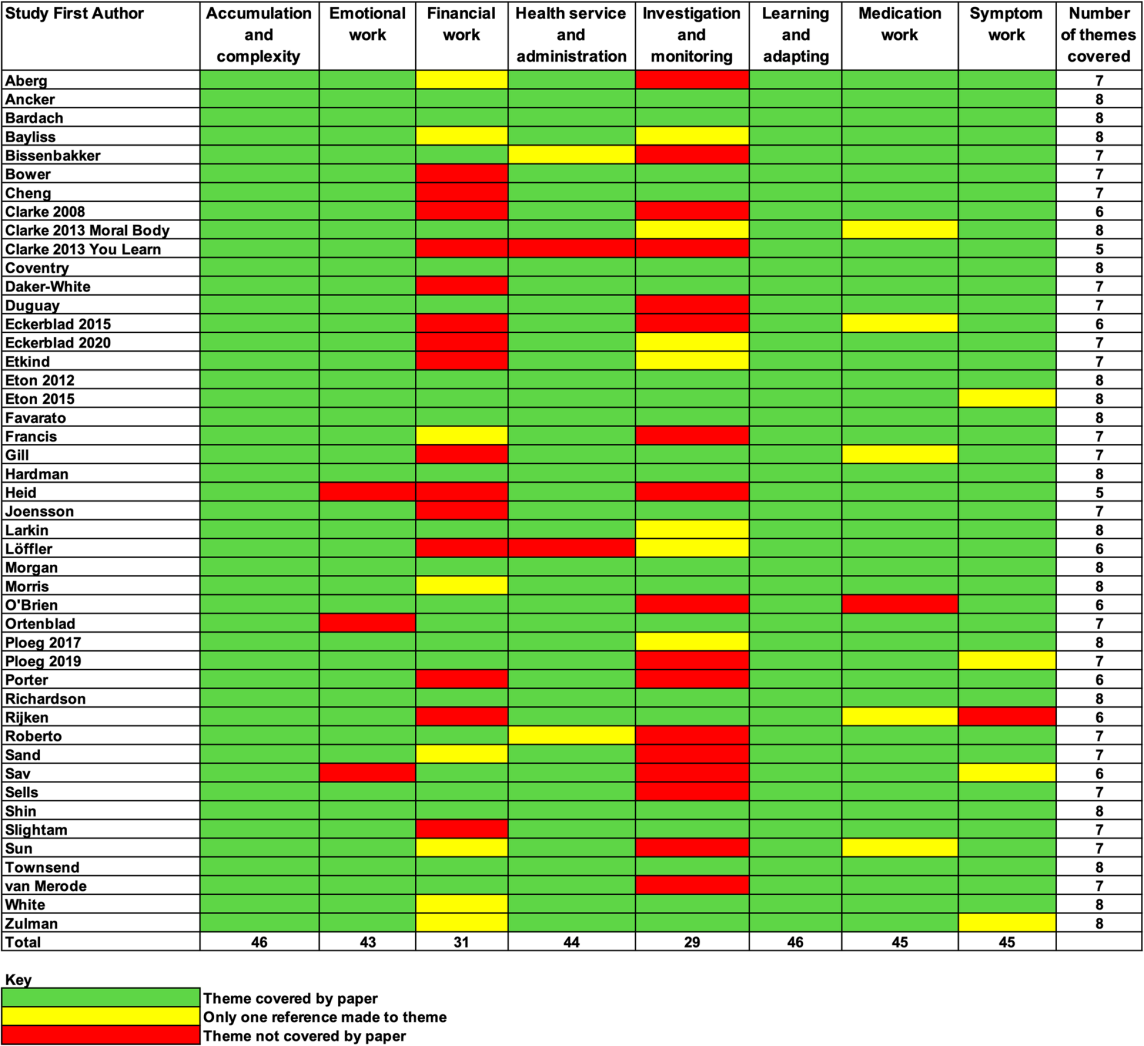
Summary of which themes of work were covered by which papers

All papers covered between five and eight of the themes (Fig. 4).

Eighteen papers covered all eight themes. Two themes (accumulating and complexity and learning and adapting) were covered by all 46 studies.

### PPI in the studies

Forty-one of the included 46 papers had no PPI involvement reported and there were no clear PPI contributor co-authors. Only one GRIPP2 reporting checklist was present (22 papers published pre 2017). Reporting of PPI did not involve public contributors, often lacked detail and provided no evidence of the process or methods used.

## Discussion

### Summary

This evidence synthesis identified that the impact of living with MLTCs is experienced as a multifaceted and complex workload summarised by eight key themes. These comprised the work of accumulation and complexity, learning and adapting, finance, medication, investigation and monitoring, health service and administration, symptoms and emotions. There was evidence that people with MLTCs do not just experience one theme of work, but multiple, and the impact of the specific lines of work are dynamic and not fixed. People with any combination of MLTCs may experience aspects of work in all eight themes, but the degree to which these are experienced is influenced by a wide range of factors that go beyond simply the particular combination of conditions concerned and are patient context specific, for example financial circumstances, ability to work and certain symptoms. There is very limited information about the experience of people with MLTCs in low- and middle-income countries. People with MLTCs are treated as somewhat homogenous populations with ethnicity reported in only a minority of studies and differences in experience between sexes, those of different socioeconomic status or ethnicity relatively under-explored. Most included papers in this review had no PPI involvement.

Although grouped together for this study, each paper included in this review studied a unique cohort with, for example, different age ranges, levels of socioeconomic status and different living environments. Certain themes, for example financial work and health service and administration, were more prominent in some studies due to either the health system context or study population, but most themes of work were experienced in all contexts. An important message in several studies was the need for a holistic approach to management, given the multidimensional and ‘whole life’ impact of living with MLTCs. This contrasts starkly with the frequently fragmented nature of health systems experienced by people living with MLTCs.

### Strengths and limitations

Our study had several strengths, one of which was the extent of PPI co-production and involving PPI colleagues living with MLTCs in line with NIHR guidance [96]. Others included the deliberately broad search strategy, the large number of papers screened for inclusion, the substantial number included for data synthesis, and representation from many different countries. We also followed the ENTREQ guideline to ensure transparent reporting and the GRADE-CERQual approach to assess the trustworthiness of our findings [34, 50].

There were also several limitations. It is possible that the use of a qualitative filter may have restricted the search, we were not able to include non-English language studies, and the second coder only undertook line by line coding for 10% of papers. To balance this, extensive discussions about codes and themes were undertaken within the study team. A further limitation is the possibility that some codes were overrepresented due to being present in both the original studies and in a review paper, or in duplicate populations within our included primary research studies. However, it is worth noting that no studies were included in more than one review paper and we did not apply any weighting to our themes.

There were several ways in which we deviated from our planned protocol, some of which we regard as strengths and some as limitations [35]. Based on feedback from PPI colleagues we refined our research questions and extended the search from primary care patients to include all patients, as we recognised that some may be exclusively or predominantly managed in secondary care. We also considered how the healthcare system currently impacts on the lived experience of MLTCs as part of the holistic experience, rather than as a standalone research question. These were strengths.

Other protocol deviations were potential limitations. Extending our exclusion criteria to omit studies with fewer than 50% of participants living with three or more LTCs may have missed some relevant insights as may the exclusion of studies of people living with only two conditions. This was, however, both a pragmatic suggestion due to the very high number of studies identified, and a deliberate decision made following PPI advice as we were particularly interested in the complexity that arises when juggling three or more LTCs. Some of our findings may therefore not be generalisable to those living with two LTCs, though we recognise that the lived experience of MLTCs is very context-specific and depends on which LTCs a person is living with.

Protocol deviations that are arguably neither strengths nor limitations include the use of CASP framework rather than CORE-Q for quality appraisal of studies and the fact that we have not yet focused on aspects related to inequalities or prevention. The MELD-B research collaboration is currently investigating which aspects of burden and complexity may be identifiable in routine primary care data and will consider these aspects during further work [33].

### Comparison with existing literature

In this review we generated many similar concepts to previous studies, including the demands imposed by managing medication and attending multiple appointments [13, 19, 25–32]. We also developed the idea of ‘work’ further by broadening the concept of impact and burden towards a more holistic model that incorporates the full experience of living with MLTCs, including emotions as distinct entities that might be experienced to widely differing degrees depending on the LTC combination and other factors. Work was the preferred term over ‘burden’ by the PPI contributors in this project as it recognises and legitimises the effort that people living with MLTCs undertake. This relates to the previous work of Hochschild who described the work of managing emotions including reference to ‘techniques of emotion work’ (these being *cognitive*, *bodily* and *expressive*) and their relation to morality and social rules [97].

Previous studies, as described above, have developed models that describe the burden for patients with LTCs including the ‘three lines of work’ model, the Cumulative Complexity Model and the Burden of Treatment framework [11–13, 67, 68]. A 2017 systematic review focussing on treatment burden among people with MLTCs included nine studies reflecting many similar concepts to our study, reflecting the complex and interacting nature of factors influencing burden [15]. A Danish population-based study of symptom burden that included 5,652 people with MLTCs found that, on average, each additional condition led to one more symptom, a third more impairment of daily activities (up to three conditions), and a third more worry about symptoms (up to three conditions) [9].

Our study generated the concepts described in these models, for example our medication work and healthcare and administration themes relate closely to aspects of burden of treatment theory, but we have added new themes to the overall experience of living with MLTCs. We generated emotional burden as an additional major area of work for people. We included symptom work as a separate theme, and also generated ‘investigation and monitoring’ as separate to other aspects of healthcare. Our ‘learning and adapting’ theme broadens out Rosbach et al.’s findings on diet and exercise and lack of knowledge concepts. [15] We additionally generated a novel ‘accumulation and complexity’ theme which describes the greater amount of work experienced by people due to having more than one LTC. This builds on the cumulative complexity model and is greater than simply the work of each separate LTC added together, arising due to issues such as interactions, unpredictability and the need for prioritisation by patients [15]. The variable nature of the work associated with MLTCs is in line with previous research in both the US and the UK demonstrating a change in treatment burden over time among people living with MLTCs [98, 99].

Our generation of unpredictability/uncertainty as part of our accumulation and complexity theme also links to Etkind et al.’s model of uncertainty for people living with advanced MLTCs. [66] The domains of ‘appraising and managing multiple illnesses’, ‘fragmented care and communication’, ‘feeling overwhelmed’ and ‘continual change’ were all concepts in our work themes [66].

Experience of MLTCs varies between different combinations of conditions but much of the work is common to many (or all). Our themes of work provide a structure that enhances previous models and frames possible approaches to solutions.

This review was not limited to the UK and this enhances its generalisability, though we recognise that some specific aspects of work were linked to the context of individual studies. For example, financial work has a higher impact in some health systems than others, such as the US (e.g. for those uninsured) and in lower income countries. However, it is important to note that financial work was expressed as an important issue in all contexts, for example in studies highlighting the costs of medications (Ghana, Australia) [67, 87], consultations (Netherlands, Belgium) [29] and costs of access (transport) (Ghana, Brazil, Canada, Australia) [69, 72, 76, 80, 81].

Relatively few studies focussed solely on people under 65, although 27 studies gave the minimum age of participants as ‘under 65’ so younger people with MLTCs were represented to some extent.

Ethnicity was not specified in the majority of papers. The socioeconomic status of participants was complicated to interpret, but seven papers appeared to focus specifically on participants from lower income environments and several others included a broad range of participants. Most papers included more women than men.

Some of the commonly included conditions in the qualitative MLTCs studies were diabetes, hypertension, osteoarthritis, depression and cardiovascular disease. Patients living with dementia were sometimes excluded and the experiences of patients with rare conditions were poorly represented. For several studies it was not completely clear which conditions were included.

### Implications for research and practice

Our evidence synthesis including eight themes adds to previous work to provide a new language of burden and work for use in future MLTCs research and practice. Consideration of the full breadth of work experienced by people living with MLTCs needs to be brought not only into research, but also into routine clinical care and health system organisation. Further work is needed on identifying how burdensome attributes might be identified in healthcare data, and how these might be applied in practice. There is also a need to better understand which aspects of work are perceived as the most burdensome by whom, and in which circumstances. Linked to this, our findings of the limited PPI involvement in MLTCs studies strongly suggests the need for greater and more transparent involvement. A GRIPP2 reporting checklist has been included for this study (Supplementary Table 4).

In the UK, the need for more joined up healthcare systems is well known. This is an almost consistent problem for patients who, not only need to attend multiple appointments for different conditions, but have the additional work of often needing to coordinate their own care and support communication between different systems. There is a paucity of evidence regarding interventions for care integration and their effectiveness [100]. Integrated Care Systems have to date shown limited evidence for the benefit in reducing patient workload and this needs to be a priority [101]. Indeed, a recent House of Lords (the upper house of UK Parliament) Integration of Primary and Community Care Committee has identified barriers to care integration and has made recommendations [102]. Continuity of care, which has been shown to be associated with reduced use of out-of-hours services, admission to hospital and mortality, also continues to be of high importance for patients with MLTCs [103].

The findings of our evidence synthesis show that people with MLTCs have to deal with many types of work across different LTCs and therefore suggest that self-management of individual conditions may add to challenges rather than provide help. There is a risk that the response to the person with MLTCs is to push the person towards greater self-management actions for all of the different conditions from which they suffer [104]. Despite the well-meaning intention of such endeavours, they may, ironically, lead to greater workload for people, and this workload is very likely to be felt differently among people with varying numbers and severity of conditions and different resources to respond [105, 106]. The current system is quick to add treatments and lifestyle actions but slower to coordinate care for people and enact deprescribing activities which could reduce the workload for people [107].

There is a need to respond at system level to reduce the workload across the themes generated. Clinical guidelines are urgently needed for people living with MLTCs who do not fit neatly into single disease frameworks. Policies are needed to assist those with MLTCs, for example with medication and travel costs. The emotional and biographical impact of MLTCs needs to be acknowledged and respected in clinical practice and research. Minimally disruptive medicine remains an essential goal [108]. Current interactions with heath system are too often fragmented and siloed [109, 110]. The sheer number of interactions is highly problematic, especially for those in employment or those who are carers [111].

Technological solutions may have a role, for example virtual appointments, coordination between specialties and patient held records, but there is a real risk that this will increase burden of treatment and widen inequalities for groups such as those with greater socioeconomic disadvantage who cannot access technology, those who are homeless, those with English not as their first language, and people with impairments such as vision and cognitive problems [94, 112].

## Conclusions

The impact of living with MLTCs is experienced as a multifaceted and complex workload involving multiple themes of work, many of which are reciprocally linked. The individual experience of living with MLTCs is determined not only by people’s unique combination of LTCs but by the health system they must navigate and their personal context. Much of this work, and the associated impact on people, may not be apparent to healthcare staff and current health services and policies are poorly equipped to meet the needs of this growing population.

## Supplementary Information

Supplementary Material 1.

## Data Availability

No datasets were generated or analysed during the current study.

## Abbreviations

CASP: Critical Appraisal Skills Programme
ENTREQ: Enhancing transparency in reporting the synthesis of qualitative research
FG: Focus group
GRADE-CERQual: Grading of Recommendations Assessment, Development, and Evaluation - Confidence in the Evidence from Reviews of Qualitative Research
IMD: Index of Multiple Deprivation
LMICs: Low- and middle-income countries
LTCs: Long-term conditions
MELD-B: Multidisciplinary Ecosystem to study Lifecourse Determinants and Prevention of Early-onset Burdensome Multimorbidity
MeSH®: Medical Subject Headings
MLTCs: Multiple long-term conditions
NIH: National Library of Medicine
NIHR: National Institute for Health Research
PPI: Patient and Public Involvement
PROSPERO: International Prospective Register of Systematic Reviews
QES: Qualitative Evidence Synthesis
SD: Standard deviation
SES: Socioeconomic status
