# Supplementary material for: The impact of living with multiple long-term conditions (multimorbidity) on everyday life – a qualitative evidence synthesis
Source: BMC Public Health. 2024 Dec 18;24:3446. doi: 10.1186/s12889-024-20763-8 (PMC11654051; doi:10.1186/s12889-024-20763-8)
Supplement: Supplementary file 1 — Supplementary Material 1. [file 12889_2024_20763_MOESM1_ESM.docx]

**Supporting Information**

**Table 1 – QES Database Searches**

**Ovid – MEDLINE and Embase (with comorbidity restriction)**

**Embase** 14524

**Medline** 7969

1

(accept* or access* or activ* or add* or adherence or ADLs or admin* or adversity or affect* or affliction or afford* or afraid or age of onset or agitat* or alone or annoy* or another* or anxiet* or anxious or attitude* or awkward or bad or bandwidth or barrier* or battle* or belief* or believ* or biographical disruption or bother* or brave or BREWS or bug bear* or bugger or burden or can* do or cant go on or capab* or capacity or car* or challeng* or chore* or comfort* or complex* or complicat* or comprehend* or concern* or confus* or connect* or constraint* or control* or convenien* or cope* or coping or cost of liv* or cross* or cumulative complexity or deal* or defeat* or demand* or depend* or depress* or deter* or diagnostic overshadowing or difficult* or dire or displease* or dissatisf* or distress or distrust or don't know where to or drain* or eas* or effect* or effort or embarrass* or emotion* or empath* or encumbrance* or energy or exhaust* or expens* or faff* or fatigue or fear* or fed up or feel* or figur* it out or forget* or forgot* or frustrat* or function* or get* on with it or grievance* or ha* no idea or happiness or happy or hard* or hassl* or help* or hop* or hurdle* or impact* or impair* or inconvenien* or inequalit* or interac* or interrupt* or irritat* or isolat* or life changing or life expectancy or life-changing or limit* or litera* or load or lonel* or los* or mak* sense or malaise or mental* or millstone or mistrust or monotonous or muddl* or multiple medications or navigat* or necess* or negativ* or neglect* or nervous or nightmare or oblig* or on hold or onus or opinion or optimist* or overwhelm* or pain* or panic or patience or patient-centered or patient-centred or perception or person-centered or person-centred or perspective or pessimistic or plan* or please* or polypharmacy or precari* or pressure* or prevent* or priorit* or problem* or psychological distress or quality of life or reassur* or relief or reliev* or rely* or requirement* or resources or responsibilit* or restrict* or risk to well-being or risk to wellbeing or sad* or satis* or scared or scary or self-care or self-determin* or shrink* or shrunk or slow or social network* or sorrow or soul destroying or stop* or strain or stress* or struggl* or stuck or suffer* or support* or tak* tablets or tether or theory of patient capacity or time or tired or together or treatment fatigue or tried or troubl* or trust* or try* or unbearable or unconfiden* or understand* or unhapp* or unpredict* or unsatis* or up to here or used to or view* or vulnerab* or wait* or work* or worr* or worse).ab,ti.

2

exp Caregiver Burden/ or quality of life/ or exp attitude to health/ or exp Emotions/ or personal satisfaction/ or self-efficacy/ or Stress, psychological/ or Psychological Distress/

3

1 or 2

4

(experience* or lived or perception* or feeling* or manag* or cope* or coping or deal* or self-manag* or challeng* or emotion* or patient* with or participant* with or people with or person* with or with at least or patient* representation*).ab,ti.

5

(living adj3 with).ab,ti.

6

Self Care/ or Self-Management/

7

4 or 5 or 6

8

(qualitative or focus group* or ethnograph* or fieldwork or field work or key informant or phenomolog* or documentary analysis or narrative synthesis or critical interpretive synthesis).ab,ti.

9

((semi-structured or semistructured or unstructured or informal or in-depth or indepth or face-to-face or structured or guide or narrative) adj2 (interview* or discussion* or questionnaire*)).ab,ti.

10

interviews as topic/ or focus groups/ or narration/ or qualitative research/

11

8 or 9 or 10

12

(multi morbid* or multimorbid*).ab,ti.

13

(polymorbidit* or poly-morbidit*).ab,ti.

14

(comorbid* or co morbid*).ab,ti.

15

(multiple comorbid* or multiple co-morbid*).ab,ti.

16

"condition count* ".ab,ti.

17

(multicondition* or multi condition*).ab,ti.

18

(multi disease* or multidisease* or multidisorder* or multi disorder*).ab,ti.

19

((multiple or concurrent) adj2 (chronic condition* or chronic health condition* or chronic health need* or disease* or disorder* or health condition* or health need* or long term condition* or long-term condition* or longterm condition* or morbid*)).ab,ti.

20

(chronic condition* or long term condition* or longterm condition*).ab,ti.

21

Multimorbidity/

22

exp Multiple Chronic Conditions/

23

Comorbidity/

24

limit 23 to yr="2000 - 2018"

25

limit 14 to yr="2000 - 2018"

26

12 or 13 or 15 or 16 or 17 or 18 or 19 or 20 or 21 or 22 or 24 or 25

27

3 and 7 and 11 and 26

28

limit 27 to yr="2000 -Current"

**EBSCO CINAHL (No comorbidity restriction)**

**CINAHL** 7746

S49

S11 AND S18 AND S27 AND S47

Limiters - Published Date: 20000101-20230131

S48

S11 AND S18 AND S27 AND S47

S47

S28 OR S29 OR S30 OR S31 OR S32 OR S33 OR S34 OR S35 OR S36 OR S37 OR S39 OR S40 OR S41 OR S42 OR S43 OR S44 OR S45

S46

(MH "Comorbidity")

S45

AB “chronic condition*” or “long term condition*” or “longterm condition*”

S44

TI “chronic condition*” or “long term condition*” or “longterm condition*”

S43

AB (multiple or concurrent) N2 (“chronic condition*” or “chronic health condition*” or “chronic health need*” or disease* or disorder* or “health condition*” or “health need*” or “long term condition*” or “long-term condition*” or “longterm condition*” or morbid*)

S42

TI (multiple or concurrent) N2 (“chronic condition*” or “chronic health condition*” or “chronic health need*” or disease* or disorder* or “health condition*” or “health need*” or “long term condition*” or “long-term condition*” or “longterm condition*” or morbid*)

S41

AB “multi disease*” or multidisease* or multidisorder* or “multi disorder*”

S40

TI “multi disease*” or multidisease* or multidisorder* or “multi disorder*”

S39

AB multicondition* or “multi condition*”

S38

TI multicondition* or “multi condition*”

S37

AB “condition count*”

S36

TI “condition count*”

S35

AB “multiple comorbid*” or “multiple co-morbid*”

S34

TI “multiple comorbid*” or “multiple co-morbid*”

S33

AB comorbid* or “co morbid*”

S32

TI comorbid* or “co morbid*”

S31

AB polymorbidit* or poly-morbidit*

S30

TI polymorbidit* or poly-morbidit*

S29

AB “multi morbid*” or multimorbid*

S28

TI “multi morbid*” or multimorbid*

S27

S19 OR S20 OR S21 OR S22 OR S23 OR S24 OR S25 OR S26

S26

(MH "Qualitative Studies")

S25

(MH "Narratives")

S24

(MH "Focus Groups")

S23

(MH "Interviews")

S22

AB (semi-structured or semistructured or unstructured or informal or in-depth or indepth or face-to-face or structured or guide or narrative) N2 (interview* or discussion* or questionnaire*)

S21

TI (semi-structured or semistructured or unstructured or informal or in-depth or indepth or face-to-face or structured or guide or narrative) N2 (interview* or discussion* or questionnaire*)

S20

AB qualitative or focus group* or ethnograph* or fieldwork or “field work” or “key informant” or phenomolog* or “documentary analysis” or “narrative synthesis” or “critical interpretive synthesis”

S19

TI qualitative or focus group* or ethnograph* or fieldwork or “field work” or “key informant” or phenomolog* or “documentary analysis” or “narrative synthesis” or “critical interpretive synthesis”

S18

S12 OR S13 OR S14 OR S15 OR S16 OR S17

S17

(MH "Self-Management")

S16

(MH "Self Care")

S15

AB living N3 with

S14

TI living N3 with

S13

AB experience* or lived or perception* or feeling* or manag* or cope* or coping or deal* or self-manag* or challeng* or emotion* or “patient* with” or “participant* with” or “people with” or “person* with” or “with at least” or “patient* representation*

S12

TI experience* or lived or perception* or feeling* or manag* or cope* or coping or deal* or self-manag* or challeng* or emotion* or “patient* with” or “participant* with” or “people with” or “person* with” or “with at least” or “patient* representation*

S11

S1 OR S2 OR S3 OR S4 OR S5 OR S6 OR S7 OR S8 OR S9 OR S10

S10

(MH "Psychological Distress")

S9

(MH "Stress, Psychological")

S8

(MH "Self-Efficacy")

S7

(MH "Personal Satisfaction")

S6

(MH "Emotions")

S5

(MH "Attitude to Health")

S4

(MH "Quality of Life")

S3

(MH "Caregiver Burden")

S2

AB (accept* or access* or activ* or add* or adherence or ADLs or admin* or adversity or affect* or affliction or afford* or afraid or “age of onset” or agitat* or alone or annoy* or another* or anxiet* or anxious or attitude* or awkward or bad or bandwidth or barrier* or battle* or belief* or believ* or “biographical disruption” or bother* or brave or BREWS or “bug bear*” or bugger or burden or “can* do” or “cant go on” or capab* or capacity or car* or challeng* or chore* or comfort* or complex* or complicat* or comprehend* or concern* or confus* or connect* or constraint* or control* or convenien* or cope* or coping or “cost of liv*” or cross* or “cumulative complexity” or deal* or defeat* or demand* or depend* or depress* or deter* or “diagnostic overshadowing” or difficult* or dire or displease* or dissatisf* or distress or distrust or “don't know where to” or drain* or eas* or effect* or effort or embarrass* or emotion* or empath* or encumbrance* or energy or exhaust* or expens* or faff* or fatigue or fear* or “fed up” or feel* or “figur* it out” or forget* or forgot* or frustrat* or function* or “get* on with it” or grievance* or “ha* no idea” or happiness or happy or hard* or hassl* or help* or hop* or hurdle* or impact* or impair* or inconvenien* or inequalit* or interac* or interrupt* or irritat* or isolat* or “life changing” or “life expectancy” or life-changing or limit* or litera* or load or lonel* or los* or “mak* sense” or malaise or mental* or millstone or mistrust or monotonous or muddl* or “multiple medications” or navigat* or necess* or negativ* or neglect* or nervous or nightmare or oblig* or “on hold” or onus or opinion or optimist* or overwhelm* or pain* or panic or patience or patient-centered or patient-centred or perception or person-centered or person-centred or perspective or pessimistic or plan* or please* or polypharmacy or precari* or pressure* or prevent* or priorit* or problem* or “psychological distress” or “quality of life” or reassur* or relief or reliev* or rely* or requirement* or resources or responsibilit* or restrict* or “risk to well-being” or “risk to wellbeing” or sad* or satis* or scared or scary or self-care or self-determin* or shrink* or shrunk or slow or “social network*” or sorrow or “soul destroying” or stop* or strain or stress* or struggl* or stuck or suffer* or support* or “tak* tablets” or tether or “theory of patient capacity” or time or tired or together or “treatment fatigue” or tried or troubl* or trust* or try* or unbearable or unconfiden* or understand* or unhapp* or unpredict* or unsatis* or “up to here” or “used to” or view* or vulnerab* or wait* or work* or worr* or worse)

S1

TI (accept* or access* or activ* or add* or adherence or ADLs or admin* or adversity or affect* or affliction or afford* or afraid or “age of onset” or agitat* or alone or annoy* or another* or anxiet* or anxious or attitude* or awkward or bad or bandwidth or barrier* or battle* or belief* or believ* or “biographical disruption” or bother* or brave or BREWS or “bug bear*” or bugger or burden or “can* do” or “cant go on” or capab* or capacity or car* or challeng* or chore* or comfort* or complex* or complicat* or comprehend* or concern* or confus* or connect* or constraint* or control* or convenien* or cope* or coping or “cost of liv*” or cross* or “cumulative complexity” or deal* or defeat* or demand* or depend* or depress* or deter* or “diagnostic overshadowing” or difficult* or dire or displease* or dissatisf* or distress or distrust or “don't know where to” or drain* or eas* or effect* or effort or embarrass* or emotion* or empath* or encumbrance* or energy or exhaust* or expens* or faff* or fatigue or fear* or “fed up” or feel* or “figur* it out” or forget* or forgot* or frustrat* or function* or “get* on with it” or grievance* or “ha* no idea” or happiness or happy or hard* or hassl* or help* or hop* or hurdle* or impact* or impair* or inconvenien* or inequalit* or interac* or interrupt* or irritat* or isolat* or “life changing” or “life expectancy” or life-changing or limit* or litera* or load or lonel* or los* or “mak* sense” or malaise or mental* or millstone or mistrust or monotonous or muddl* or “multiple medications” or navigat* or necess* or negativ* or neglect* or nervous or nightmare or oblig* or “on hold” or onus or opinion or optimist* or overwhelm* or pain* or panic or patience or patient-centered or patient-centred or perception or person-centered or person-centred or perspective or pessimistic or plan* or please* or polypharmacy or precari* or pressure* or prevent* or priorit* or problem* or “psychological distress” or “quality of life” or reassur* or relief or reliev* or rely* or requirement* or resources or responsibilit* or restrict* or “risk to well-being” or “risk to wellbeing” or sad* or satis* or scared or scary or self-care or self-determin* or shrink* or shrunk or slow or “social network*” or sorrow or “soul destroying” or stop* or strain or stress* or struggl* or stuck or suffer* or support* or “tak* tablets” or tether or “theory of patient capacity” or time or tired or together or “treatment fatigue” or tried or troubl* or trust* or try* or unbearable or unconfiden* or understand* or unhapp* or unpredict* or unsatis* or “up to here” or “used to” or view* or vulnerab* or wait* or work* or worr* or worse)

**EBSCO PsycInfo (No comorbidity restriction)**

PsycInfo 6616

PsycArticles 104

S48

S10 AND S17 AND S26 AND S46

Limiters - Published Date: 20000101-20230131

S47

S10 AND S17 AND S26 AND S46

S46

S27 OR S28 OR S29 OR S30 OR S31 OR S32 OR S33 OR S34 OR S35 OR S36 OR S37 OR S38 OR S39 OR S40 OR S41 OR S42 OR S43 OR S44 OR S45

S45

DE "Comorbidity"

S44

AB “chronic condition*” or “long term condition*” or “longterm condition*”

S43

TI “chronic condition*” or “long term condition*” or “longterm condition*”

S42

AB (multiple or concurrent) N2 (“chronic condition*” or “chronic health condition*” or “chronic health need*” or disease* or disorder* or “health condition*” or “health need*” or “long term condition*” or “long-term condition*” or “longterm condition*” or morbid*)

S41

TI (multiple or concurrent) N2 (“chronic condition*” or “chronic health condition*” or “chronic health need*” or disease* or disorder* or “health condition*” or “health need*” or “long term condition*” or “long-term condition*” or “longterm condition*” or morbid*)

S40

AB “multi disease*” or multidisease* or multidisorder* or “multi disorder*”

S39

TI “multi disease*” or multidisease* or multidisorder* or “multi disorder*”

S38

AB multicondition* or “multi condition*”

S37

TI multicondition* or “multi condition*”

S36

AB “condition count*”

S35

TI “condition count*”

S34

AB “multiple comorbid*” or “multiple co-morbid*”

S33

TI “multiple comorbid*” or “multiple co-morbid*”

S32

AB comorbid* or “co morbid*”

S31

TI comorbid* or “co morbid*”

S30

AB polymorbidit* or poly-morbidit*

S29

TI polymorbidit* or poly-morbidit*

S28

AB “multi morbid*” or multimorbid*

S27

TI “multi morbid*” or multimorbid*

S26

S18 OR S19 OR S20 OR S21 OR S22 OR S24 OR S25

S25

DE "Qualitative Methods" OR DE "Qualitative Measures"

S24

DE "Narrative Analysis" OR DE "Narratives"

S23

DE "Focus Group Interview" OR DE "Focus Group"

S22

DE "Interviews"

S21

AB (semi-structured or semistructured or unstructured or informal or in-depth or indepth or face-to-face or structured or guide or narrative) N2 (interview* or discussion* or questionnaire*)

S20

TI (semi-structured or semistructured or unstructured or informal or in-depth or indepth or face-to-face or structured or guide or narrative) N2 (interview* or discussion* or questionnaire*)

S19

AB qualitative or focus group* or ethnograph* or fieldwork or “field work” or “key informant” or phenomolog* or “documentary analysis” or “narrative synthesis” or “critical interpretive synthesis”

S18

TI qualitative or focus group* or ethnograph* or fieldwork or “field work” or “key informant” or phenomolog* or “documentary analysis” or “narrative synthesis” or “critical interpretive synthesis”

S17

S11 OR S12 OR S13 OR S14 OR S15 OR S16

S16

DE "Self-Management"

S15

DE "Self-Care"

S14

AB living N3 with

S13

TI living N3 with

S12

AB experience* or lived or perception* or feeling* or manag* or cope* or coping or deal* or self-manag* or challeng* or emotion* or “patient* with” or “participant* with” or “people with” or “person* with” or “with at least” or “patient* representation*”

S11

TI experience* or lived or perception* or feeling* or manag* or cope* or coping or deal* or self-manag* or challeng* or emotion* or “patient* with” or “participant* with” or “people with” or “person* with” or “with at least” or “patient* representation*”

S10

S1 OR S2 OR S3 OR S4 OR S5 OR S6 OR S7 OR S8 OR S9

S9

DE "Psychological Stress"

S8

DE "Self-Efficacy"

S7

DE "Subjective Well Being"

S6

DE "Emotions"

S5

DE "Health Attitudes"

S4

DE "Quality of Life"

S3

DE "Caregiver Burden"

S2

AB (accept* or access* or activ* or add* or adherence or ADLs or admin* or adversity or affect* or affliction or afford* or afraid or “age of onset” or agitat* or alone or annoy* or another* or anxiet* or anxious or attitude* or awkward or bad or bandwidth or barrier* or battle* or belief* or believ* or “biographical disruption” or bother* or brave or BREWS or “bug bear*” or bugger or burden or “can* do” or “cant go on” or capab* or capacity or car* or challeng* or chore* or comfort* or complex* or complicat* or comprehend* or concern* or confus* or connect* or constraint* or control* or convenien* or cope* or coping or “cost of liv*” or cross* or “cumulative complexity” or deal* or defeat* or demand* or depend* or depress* or deter* or “diagnostic overshadowing” or difficult* or dire or displease* or dissatisf* or distress or distrust or “don't know where to” or drain* or eas* or effect* or effort or embarrass* or emotion* or empath* or encumbrance* or energy or exhaust* or expens* or faff* or fatigue or fear* or “fed up” or feel* or “figur* it out” or forget* or forgot* or frustrat* or function* or “get* on with it” or grievance* or “ha* no idea” or happiness or happy or hard* or hassl* or help* or hop* or hurdle* or impact* or impair* or inconvenien* or inequalit* or interac* or interrupt* or irritat* or isolat* or “life changing” or “life expectancy” or life-changing or limit* or litera* or load or lonel* or los* or “mak* sense” or malaise or mental* or millstone or mistrust or monotonous or muddl* or “multiple medications” or navigat* or necess* or negativ* or neglect* or nervous or nightmare or oblig* or “on hold” or onus or opinion or optimist* or overwhelm* or pain* or panic or patience or patient-centered or patient-centred or perception or person-centered or person-centred or perspective or pessimistic or plan* or please* or polypharmacy or precari* or pressure* or prevent* or priorit* or problem* or “psychological distress” or “quality of life” or reassur* or relief or reliev* or rely* or requirement* or resources or responsibilit* or restrict* or “risk to well-being” or “risk to wellbeing” or sad* or satis* or scared or scary or self-care or self-determin* or shrink* or shrunk or slow or “social network*” or sorrow or “soul destroying” or stop* or strain or stress* or struggl* or stuck or suffer* or support* or “tak* tablets” or tether or “theory of patient capacity” or time or tired or together or “treatment fatigue” or tried or troubl* or trust* or try* or unbearable or unconfiden* or understand* or unhapp* or unpredict* or unsatis* or “up to here” or “used to” or view* or vulnerab* or wait* or work* or worr* or worse)

S1

TI (accept* or access* or activ* or add* or adherence or ADLs or admin* or adversity or affect* or affliction or afford* or afraid or “age of onset” or agitat* or alone or annoy* or another* or anxiet* or anxious or attitude* or awkward or bad or bandwidth or barrier* or battle* or belief* or believ* or “biographical disruption” or bother* or brave or BREWS or “bug bear*” or bugger or burden or “can* do” or “cant go on” or capab* or capacity or car* or challeng* or chore* or comfort* or complex* or complicat* or comprehend* or concern* or confus* or connect* or constraint* or control* or convenien* or cope* or coping or “cost of liv*” or cross* or “cumulative complexity” or deal* or defeat* or demand* or depend* or depress* or deter* or “diagnostic overshadowing” or difficult* or dire or displease* or dissatisf* or distress or distrust or “don't know where to” or drain* or eas* or effect* or effort or embarrass* or emotion* or empath* or encumbrance* or energy or exhaust* or expens* or faff* or fatigue or fear* or “fed up” or feel* or “figur* it out” or forget* or forgot* or frustrat* or function* or “get* on with it” or grievance* or “ha* no idea” or happiness or happy or hard* or hassl* or help* or hop* or hurdle* or impact* or impair* or inconvenien* or inequalit* or interac* or interrupt* or irritat* or isolat* or “life changing” or “life expectancy” or life-changing or limit* or litera* or load or lonel* or los* or “mak* sense” or malaise or mental* or millstone or mistrust or monotonous or muddl* or “multiple medications” or navigat* or necess* or negativ* or neglect* or nervous or nightmare or oblig* or “on hold” or onus or opinion or optimist* or overwhelm* or pain* or panic or patience or patient-centered or patient-centred or perception or person-centered or person-centred or perspective or pessimistic or plan* or please* or polypharmacy or precari* or pressure* or prevent* or priorit* or problem* or “psychological distress” or “quality of life” or reassur* or relief or reliev* or rely* or requirement* or resources or responsibilit* or restrict* or “risk to well-being” or “risk to wellbeing” or sad* or satis* or scared or scary or self-care or self-determin* or shrink* or shrunk or slow or “social network*” or sorrow or “soul destroying” or stop* or strain or stress* or struggl* or stuck or suffer* or support* or “tak* tablets” or tether or “theory of patient capacity” or time or tired or together or “treatment fatigue” or tried or troubl* or trust* or try* or unbearable or unconfiden* or understand* or unhapp* or unpredict* or unsatis* or “up to here” or “used to” or view* or vulnerab* or wait* or work* or worr* or worse)

**Cochrane**

Used Chrome and accepted cookies so that the export function worked

Did not apply comorbidity date restriction as difficult to do so using the interface

Cochrane Reviews 725

Cochrane Protocols 2

Trials 7926

Editorials 20 – could not export

ID Search Hits

#1 (accept* or access* or activ* or add* or adherence or ADLs or admin* or adversity or affect* or affliction or afford* or afraid or age of onset or agitat* or alone or annoy* or another* or anxiet* or anxious or attitude* or awkward or bad or bandwidth or barrier* or battle* or belief* or believ* or biographical disruption or bother* or brave or BREWS or bug bear* or bugger or burden or can* do or cant go on or capab* or capacity or car* or challeng* or chore* or comfort* or complex* or complicat* or comprehend* or concern* or confus* or connect* or constraint* or control* or convenien* or cope* or coping or cost of liv* or cross* or cumulative complexity or deal* or defeat* or demand* or depend* or depress* or deter* or diagnostic overshadowing or difficult* or dire or displease* or dissatisf* or distress or distrust or don't know where to or drain* or eas* or effect* or effort or embarrass* or emotion* or empath* or encumbrance* or energy or exhaust* or expens* or faff* or fatigue or fear* or fed up or feel* or figur* it out or forget* or forgot* or frustrat* or function* or get* on with it or grievance* or ha* no idea or happiness or happy or hard* or hassl* or help* or hop* or hurdle* or impact* or impair* or inconvenien* or inequalit* or interac* or interrupt* or irritat* or isolat* or life changing or life expectancy or life-changing or limit* or litera* or load or lonel* or los* or mak* sense or malaise or mental* or millstone or mistrust or monotonous or muddl* or multiple medications or navigat* or necess* or negativ* or neglect* or nervous or nightmare or oblig* or on hold or onus or opinion or optimist* or overwhelm* or pain* or panic or patience or patient-centered or patient-centred or perception or person-centered or person-centred or perspective or pessimistic or plan* or please* or polypharmacy or precari* or pressure* or prevent* or priorit* or problem* or psychological distress or quality of life or reassur* or relief or reliev* or rely* or requirement* or resources or responsibilit* or restrict* or risk to well-being or risk to wellbeing or sad* or satis* or scared or scary or self-care or self-determin* or shrink* or shrunk or slow or social network* or sorrow or soul destroying or stop* or strain or stress* or struggl* or stuck or suffer* or support* or tak* tablets or tether or theory of patient capacity or time or tired or together or treatment fatigue or tried or troubl* or trust* or try* or unbearable or unconfiden* or understand* or unhapp* or unpredict* or unsatis* or up to here or used to or view* or vulnerab* or wait* or work* or worr* or worse):ti,ab,kw (Word variations have been searched)

#2 MeSH descriptor: [Caregiver Burden] explode all trees

#3 MeSH descriptor: [Quality of Life] explode all trees

#4 MeSH descriptor: [Attitude to Health] explode all trees

#5 MeSH descriptor: [Emotions] explode all trees

#6 MeSH descriptor: [Personal Satisfaction] explode all trees

#7 MeSH descriptor: [Self Efficacy] explode all trees

#8 MeSH descriptor: [Stress, Psychological] explode all trees

#9 MeSH descriptor: [Psychological Distress] explode all trees

#10 #1 OR #2 OR #3 OR #4 OR #5 OR #6 OR #7 OR #8 OR #9

#11 (experience* or lived or perception* or feeling* or manag* or cope* or coping or deal* or self-manag* or challeng* or emotion* or patient* with or participant* with or people with or person* with or with at least or patient* representation*):ti,ab,kw (Word variations have been searched)

#12 (living NEAR/3 with):ti,ab,kw

#13 MeSH descriptor: [Self Care] explode all trees

#14 MeSH descriptor: [Self-Management] explode all trees

#15 #11 OR #12 OR #13 OR #14

#16 (qualitative or focus group* or ethnograph* or fieldwork or field work or key informant or phenomolog* or documentary analysis or narrative synthesis or critical interpretive synthesis):ti,ab,kw (Word variations have been searched)

#17 ((semi-structured or semistructured or unstructured or informal or in-depth or indepth or face-to-face or structured or guide or narrative) NEAR/2 (interview* or discussion* or questionnaire*)):ti,ab,kw

#18 MeSH descriptor: [Interview] explode all trees

#19 MeSH descriptor: [Focus Groups] explode all trees

#20 MeSH descriptor: [Narration] explode all trees

#21 MeSH descriptor: [Qualitative Research] explode all trees

#22 #16 OR #17 OR #18 OR #19 OR #20 OR #21

#23 (multi morbid* or multimorbid*):ti,ab,kw (Word variations have been searched)

#24 (polymorbidit* or poly-morbidit*):ti,ab,kw (Word variations have been searched)

#25 (comorbid* or co morbid*):ti,ab,kw (Word variations have been searched)

#26 (multiple comorbid* or multiple co-morbid*):ti,ab,kw (Word variations have been searched)

#27 ("condition count*"):ti,ab,kw (Word variations have been searched)

#28 (multicondition* or multi condition*):ti,ab,kw (Word variations have been searched)

#29 (multi disease* or multidisease* or multidisorder* or multi disorder*):ti,ab,kw (Word variations have been searched)

#30 ((multiple or concurrent) NEAR/2 (chronic condition* or chronic health condition* or chronic health need* or disease* or disorder* or health condition* or health need* or long term condition* or long-term condition* or longterm condition* or morbid*)):ti,ab,kw

#31 (chronic condition* or long term condition* or longterm condition*):ti,ab,kw (Word variations have been searched)

#32 MeSH descriptor: [Multimorbidity] explode all trees

#33 MeSH descriptor: [Multiple Chronic Conditions] explode all trees

#34 MeSH descriptor: [Comorbidity] explode all trees

#35 #23 OR #24 OR #25 OR #26 OR #27 OR #28 OR #29 OR #30 OR #31 OR #32 OR #33 OR #3

#36 #10 AND #15 AND #22 AND #35

**Supplementary table 2 - ENTREQ Checklist**

| Number | Item | Guide and description | Details | Page of report |
| --- | --- | --- | --- | --- |
| 1 | Aim | State the research question the synthesis addresses. | What is it like to live with MLTCs (multimorbidity) and which aspects do people living with MLTCs consider burdensome and make living with multimorbidity complex?  Was there any PPI input into the papers identified by this review? | 7 |
| 2 | Synthesis methodology | Identify the synthesis methodology or theoretical framework which underpins the synthesis, and describe the rationale for choice of methodology *(e.g. meta-ethnography, thematic synthesis, critical interpretive synthesis, grounded theory synthesis, realist synthesis, meta-aggregation, meta-study, framework synthesis).* | Thematic synthesis | 11 |
| 3 | Approach to searching | Indicate whether the search was pre-planned (*comprehensive search strategies to seek all available studies)* or iterative (*to seek all available concepts until they theoretical saturation is achieved)*. | Pre-planned comprehensive search | 9 |
| 4 | Inclusion criteria | Specify the inclusion/exclusion criteria *(e.g. in terms of population, language, year limits, type of publication, study type).* | Inclusion Criteria:   - Studies involving papers where at least 50% of participants with living with 3 or more long term conditions - A focus on multimorbidity (not a focus on one or two conditions with comorbidity) - Studies exploring lived experience of multiple long-term conditions from the point of view of patients - Qualitative studies (primary research or qualitative syntheses) and mixed methods studies with a relevant qualitative component - All settings including home and other community settings (including private, rented, social housing, care home, prisons, homeless) and clinical settings (primary care, secondary care, intermediate care, etc.)   Exclusion criteria:   - Studies involving papers where fewer than 50% of participants were living with 3 or more LTCs - Studies focussing on one or two clear index conditions and comorbidities - (This allowed a greater focus on multimorbidity rather than co-morbidity and was also a pragmatic decision due to the very high number of studies identified by the criteria of two or more LTCs) - Not the patient perspective - Quantitative studies (except mixed methods studies with a substantial qualitative component) - Studies including children - Not in English - Conference abstract (no full text article) - Duplicate (the same study with more than one record in Rayyan) - Studies with a focus on medicines - Studies with a focus on self-management (helping people manage better) - Studies with a focus on the use of technology (e.g. patients’ views on telehealth) - Studies with a focus on interventions | 7 |
| 5 | Data sources | Describe the information sources used (e.g. *electronic databases (MEDLINE, EMBASE, CINAHL, psycINFO, Econlit), grey literature databases (digital thesis, policy reports), relevant organisational websites, experts, information specialists, generic web searches (Google Scholar) hand searching, reference lists)* and when the searches conducted; provide the rationale for using the data sources. | MEDLINE (Ovid), EMBASE (Ovid), PsycINFO (EBSCO), PsycArticles (EBSCO), CINAHL (EBSCO), the Cochrane Library  The Journal of Multimorbidity and Comorbidity was manually searched  Reference list searching from included articles and follow up with authors as needed. | 9 |
| 6 | Electronic Search strategy | Describe the literature search *(e.g. provide electronic search strategies with population terms, clinical or health topic terms, experiential or social phenomena related terms, filters for qualitative research, and search limits)*. | Search terms were developed as three concepts (multimorbidity, burden and lived experience) with a qualitative filter, each with a string of terms and relevant MeSH terms, and were developed from a review of grey literature reports, published searches, PhD/MD theses and an online thesaurus.  The searches were conducted in January 2023 and the date range was restricted to 1^st^ January 2000 onwards for pragmatic reasons (the very high number of studies). The date range for the term ‘comorbidity’ was restricted from 2000-2018 in MEDLINE and Embase as this term was used prior to the introduction of the Medical Subject Headings (MeSH) terms ‘multiple chronic conditions’ and ‘multiple long-term conditions’ by National Library of Medicine (NIH) in 2017 and 2018 respectively. | 9 |
| 7 | Study screening methods | Describe the process of study screening and sifting *(e.g. title, abstract and full text review, number of independent reviewers who screened studies).* | Double screening of the title/abstract and subsequently the full text of studies was conducted by two researchers independently (blinded)  Discrepancies in both title/abstract and full text screening were adjudicated by a third researcher. Studies were not excluded based on quality. | 10 |
| 8 | Study characteristics | Present the characteristics of the included studies *(e.g. year of publication, country, population, number of participants, data collection, methodology, analysis, research questions).* | Table 2 contains the following information:  Study first author  Year of publication  Location  Data collection method  Number of participants  Age of participants  Average age (mean unless otherwise specified)  Sex of participants  Ethnicity of participants  Socioeconomic status of participants (SES)  Number of conditions | 31-32 and Table 2 |
| 9 | Study selection results | Identify the number of studies screened and provide reasons for study exclusion *(e,g, for comprehensive searching, provide numbers of studies screened and reasons for exclusion indicated in a figure/flowchart; for iterative searching describe reasons for study exclusion and inclusion based on modifications t the research question and/or contribution to theory development).* | 30803 unique studies were screened  Reasons were not recorded for exclusions at title/abstract level (due to very high numbers)  Exclusion reasons at full text screening (n = 72), also see Figure 1:  Fewer than 3 conditions (n=28)  Not work or burden (n=14)  Abstract (n=9)  Focus on self-management (n=8)  Not qualitative research 5 (n=5)  Duplicate (n=3)  Focus on medication (n=2)  Not lived experience (n=2)  Not patient perspective (n=1) | 14-15 and Figure 1 |
| 10 | Rationale for appraisal | Describe the rationale and approach used to appraise the included studies or selected findings *(e.g. assessment of conduct (validity and robustness), assessment of reporting (transparency), assessment of content and utility of the findings).* | We used the Critical Appraisal Skills Program (CASP) check list for qualitative research. This decision was based on the lead author’s limited experience of quality assessment of qualitative studies and following the Cochrane training handbook Chapter 21 section 8.  <https://training.cochrane.org/handbook/current/chapter-21> | 10 |
| 11 | Appraisal items | State the tools, frameworks and criteria used to appraise the studies or selected findings *(e.g. Existing tools: CASP, QARI, COREQ, Mays and Pope* [[25](https://bmcmedresmethodol.biomedcentral.com/articles/10.1186/1471-2288-12-181#ref-CR25)]*; reviewer developed tools; describe the domains assessed: research team, study design, data analysis and interpretations, reporting).* | Critical Appraisal Skills Programme checklist for qualitative research | 10 |
| 12 | Appraisal process | Indicate whether the appraisal was conducted independently by more than one reviewer and if consensus was required. | 2 researchers (EH and one of KSYC, SS, MA, CG, SF) independently undertook the quality assessment of included studies (blinded). In any cases where there was initial disagreement between the two assessors on an aspect of study quality then a conservative approach was adopted and the lower quality category was chosen. | 10 |
| 13 | Appraisal results | Present results of the quality assessment and indicate which articles, if any, were weighted/excluded based on the assessment and give the rationale. | Please see Figure 2  No studies were excluded based on this assessment and no weighting was applied | 32-33 |
| 14 | Data extraction | Indicate which sections of the primary studies were analysed and how were the data extracted from the primary studies? *(e.g. all text under the headings “results /conclusions” were extracted electronically and entered into a computer software).* | Paper PDFs were converted into word documents and imported into NVivo. All text relating to burden in Results and Discussion sections of papers were coded, except where information was not from the patient perspective, for example in studies who also interviewed spouses, caregivers and healthcare providers. | 10 |
| 15 | Software | State the computer software used, if any. | Endnote was used to store references  Rayyan software and Microsoft Excel were used for screening  NVivo was used for coding | 9-11 |
| 16 | Number of reviewers | Identify who was involved in coding and analysis. | Screening – EH, KM, KSYC, LL, MA, SF  Quality assessment – EH, KSYC, SS, MA, CG and SF  Coding – EH and SF  Analysis of PPI input to the studies - LL  Analysis discussions – EH, SF, PPI co-author LL and subject experts FM, SM, MA, NF | 9-13 |
| 17 | Coding | Describe the process for coding of data *(e.g. line by line coding to search for concepts).* | Line by line coding was undertaken in NVivo for all studies by EH. A second coder (SF) manually coded 10% (5 papers) and coding was compared for agreement, with no new codes being identified. Regular lengthy and active discussions took place between EH, SF and subject expert SM over the course of the analysis. | 11 |
| 18 | Study comparison | Describe how were comparisons made within and across studies *(e.g. subsequent studies were coded into pre-existing concepts, and new concepts were created when deemed necessary).* | A ‘constant comparison’ approach to discussions was taken whereby codes and emerging themes were repeatedly discussed and iterated over several months within the research team (including PPI coauthor LL and subject experts FM, MA, NF, SF, SM), the wider MELD-B team, and with the wider MELD-B PPI Advisory Board to check for relevance and understanding. | 11 |
| 19 | Derivation of themes | Explain whether the process of deriving the themes or constructs was inductive or deductive. | An inductive approach was taken | 3 |
| 20 | Quotations | Provide quotations from the primary studies to illustrate themes/constructs and identify whether the quotations were participant quotations or the author’s interpretation. | Please see Table 3 and Results sections | 35-66 |
| 21 | Synthesis output | Present rich, compelling and useful results that go beyond a summary of the primary studies (e.g. *new interpretation, models of evidence, conceptual models, analytical framework, development of a new theory or construct).* | This evidence synthesis identified that the impact of living with MLTCs is experienced as a multifaceted and complex workload summarised by eight key themes. These comprised the work of accumulation and complexity, learning and adapting, finance, medication, investigation and monitoring, health service and administration, symptoms and emotions.  This adds to previous work to provide a new language of burden and work for use in future MLTCs research and practice. | 68, 73-74 |

**Supplementary Table 3 – GRADE-CERQual Assessment**

**The burden of living with multimorbidity**

**Summary of Qualitative Findings Table**

**Review question**

What features of living with multimorbidity do patients consider burdensome?

**Authors of the review**

Emilia Holland, Kate Matthews, Sara MacDonald, Lynn Laidlaw, Rita Rajababoo, Saroj Parekh, Kelly Cheung, Seb Stannard, Mark Ashworth, Nick Francis, Frances Mair, Nisreen Alwan, Simon DS Fraser

**Corresponding author**

Simon Fraser - s.fraser@soton.ac.uk

**Has the review been published?**

No

**Additional Information**

This review is part of the MELD-B project funded by the NIHR AIM programme

| **#** | **Summarised review finding** | **GRADE-CERQual Assessment of confidence** | **Explanation of GRADE-CERQual Assessment** | **References** |
| --- | --- | --- | --- | --- |
| 1 | People living with MLTCs do not just experience one type of work, but multiple, and these occur in differing combinations depending on the nature and combination of conditions and other factors. | High confidence | No/Very minor concerns regarding methodological limitations, No/Very minor concerns regarding coherence, No/Very minor concerns regarding adequacy, and No/Very minor concerns regarding relevance | Bayliss EA et al. 2003; Clarke LH et al. 2008; Cheng C et al. 2019; Bower P et al. 2012; Bissenbakker K et al. 2022; Åberg C et al. 2020; Clarke LH & Bennett EV 2013; Clarke LH & Bennett E 2013; Bardach SH et al. 2011; Favarato MH et al. 2021; Francis H et al. 2020; Eckerblad J et al. 2015; Eckerblad J et al. 2020; Coventry PA et al. 2015; Duguay C et al. 2014; Eton DT et al. 2012; Heid AR et al. 2020; Hardman R et al. 2021; Ørtenblad L et al. 2018; Löffler C et al. 2012; Morgan SA et al. 2019; O'Brien R et al. 2014; Gill A et al. 2014; Morris RL et al. 2011; Joensson ABR et al. 2020; Larkin J et al. 2021; Shin JW et al. 2022; Roberto KA et al. 2005; Sav A et al. 2013; Sand CD et al. 2021; Sells D et al. 2009; Porter T et al. 2020; van Merode T et al. 2018; White C et al. 2016; Zulman DM et al. 2015; Sun L et al. 2022; Townsend A et al. 2008; Ancker JS et al. 2015; Eton DT et al. 2015; Daker-White G et al. 2018; Etkind SN et al. 2022; Richardson LM et al. 2016; Ploeg J et al. 2017; Ploeg J et al. 2019; Slightam CA et al. 2018; |
| 2 | Symptom work as a theme of burden | High confidence | No/Very minor concerns regarding methodological limitations, No/Very minor concerns regarding coherence, No/Very minor concerns regarding adequacy, and No/Very minor concerns regarding relevance | Bayliss EA et al. 2003; Clarke LH et al. 2008; Cheng C et al. 2019; Bower P et al. 2012; Bissenbakker K et al. 2022; Åberg C et al. 2020; Clarke LH & Bennett EV 2013; Clarke LH & Bennett E 2013; Bardach SH et al. 2011; Favarato MH et al. 2021; Francis H et al. 2020; Eckerblad J et al. 2015; Eckerblad J et al. 2020; Coventry PA et al. 2015; Duguay C et al. 2014; Eton DT et al. 2012; Heid AR et al. 2020; Hardman R et al. 2021; Ørtenblad L et al. 2018; Löffler C et al. 2012; Morgan SA et al. 2019; O'Brien R et al. 2014; Gill A et al. 2014; Morris RL et al. 2011; Joensson ABR et al. 2020; Larkin J et al. 2021; Shin JW et al. 2022; Roberto KA et al. 2005; Sav A et al. 2013; Sand CD et al. 2021; Sells D et al. 2009; Rijken M et al. 2021; Porter T et al. 2020; van Merode T et al. 2018; White C et al. 2016; Zulman DM et al. 2015; Sun L et al. 2022; Townsend A et al. 2008; Ancker JS et al. 2015; Eton DT et al. 2015; Daker-White G et al. 2018; Etkind SN et al. 2022; Richardson LM et al. 2016; Ploeg J et al. 2017; Ploeg J et al. 2019; Slightam CA et al. 2018; |
| 3 | Emotional work as a theme of burden | High confidence | No/Very minor concerns regarding methodological limitations, No/Very minor concerns regarding coherence, No/Very minor concerns regarding adequacy, and No/Very minor concerns regarding relevance | Bayliss EA et al. 2003; Clarke LH et al. 2008; Cheng C et al. 2019; Bower P et al. 2012; Bissenbakker K et al. 2022; Åberg C et al. 2020; Clarke LH & Bennett EV 2013; Clarke LH & Bennett E 2013; Bardach SH et al. 2011; Favarato MH et al. 2021; Francis H et al. 2020; Eckerblad J et al. 2015; Eckerblad J et al. 2020; Coventry PA et al. 2015; Duguay C et al. 2014; Eton DT et al. 2012; Heid AR et al. 2020; Hardman R et al. 2021; Ørtenblad L et al. 2018; Löffler C et al. 2012; Morgan SA et al. 2019; O'Brien R et al. 2014; Gill A et al. 2014; Morris RL et al. 2011; Joensson ABR et al. 2020; Larkin J et al. 2021; Shin JW et al. 2022; Roberto KA et al. 2005; Sav A et al. 2013; Sand CD et al. 2021; Sells D et al. 2009; Rijken M et al. 2021; Porter T et al. 2020; van Merode T et al. 2018; White C et al. 2016; Zulman DM et al. 2015; Sun L et al. 2022; Townsend A et al. 2008; Ancker JS et al. 2015; Eton DT et al. 2015; Daker-White G et al. 2018; Etkind SN et al. 2022; Richardson LM et al. 2016; Ploeg J et al. 2017; Ploeg J et al. 2019; Slightam CA et al. 2018; |
| 4 | Investigation and monitoring work as a theme of burden | High confidence | No/Very minor concerns regarding methodological limitations, No/Very minor concerns regarding coherence, No/Very minor concerns regarding adequacy, and No/Very minor concerns regarding relevance | Bayliss EA et al. 2003; Cheng C et al. 2019; Bower P et al. 2012; Clarke LH & Bennett EV 2013; Bardach SH et al. 2011; Favarato MH et al. 2021; Eckerblad J et al. 2020; Coventry PA et al. 2015; Eton DT et al. 2012; Hardman R et al. 2021; Ørtenblad L et al. 2018; Löffler C et al. 2012; Morgan SA et al. 2019; Gill A et al. 2014; Morris RL et al. 2011; Joensson ABR et al. 2020; Larkin J et al. 2021; Shin JW et al. 2022; Rijken M et al. 2021; Porter T et al. 2020; White C et al. 2016; Zulman DM et al. 2015; Townsend A et al. 2008; Ancker JS et al. 2015; Eton DT et al. 2015; Daker-White G et al. 2018; Etkind SN et al. 2022; Richardson LM et al. 2016; Ploeg J et al. 2017; Slightam CA et al. 2018; |
| 5 | Health service and administration work as a theme of burden | High confidence | No/Very minor concerns regarding methodological limitations, No/Very minor concerns regarding coherence, No/Very minor concerns regarding adequacy, and No/Very minor concerns regarding relevance | Bayliss EA et al. 2003; Clarke LH et al. 2008; Cheng C et al. 2019; Bower P et al. 2012; Bissenbakker K et al. 2022; Åberg C et al. 2020; Clarke LH & Bennett EV 2013; Clarke LH & Bennett E 2013; Bardach SH et al. 2011; Favarato MH et al. 2021; Francis H et al. 2020; Eckerblad J et al. 2015; Eckerblad J et al. 2020; Coventry PA et al. 2015; Duguay C et al. 2014; Eton DT et al. 2012; Heid AR et al. 2020; Hardman R et al. 2021; Ørtenblad L et al. 2018; Löffler C et al. 2012; Morgan SA et al. 2019; O'Brien R et al. 2014; Gill A et al. 2014; Morris RL et al. 2011; Joensson ABR et al. 2020; Larkin J et al. 2021; Shin JW et al. 2022; Roberto KA et al. 2005; Sav A et al. 2013; Sand CD et al. 2021; Sells D et al. 2009; Rijken M et al. 2021; Porter T et al. 2020; van Merode T et al. 2018; Sun L et al. 2022; Townsend A et al. 2008; Ancker JS et al. 2015; Eton DT et al. 2015; Daker-White G et al. 2018; Etkind SN et al. 2022; Richardson LM et al. 2016; Ploeg J et al. 2017; Ploeg J et al. 2019; Slightam CA et al. 2018; |
| 6 | Medication work as a theme of burden | High confidence | No/Very minor concerns regarding methodological limitations, No/Very minor concerns regarding coherence, No/Very minor concerns regarding adequacy, and No/Very minor concerns regarding relevance | Bayliss EA et al. 2003; Clarke LH et al. 2008; Cheng C et al. 2019; Bower P et al. 2012; Bissenbakker K et al. 2022; Åberg C et al. 2020; Clarke LH & Bennett EV 2013; Clarke LH & Bennett E 2013; Bardach SH et al. 2011; Favarato MH et al. 2021; Francis H et al. 2020; Eckerblad J et al. 2015; Eckerblad J et al. 2020; Coventry PA et al. 2015; Duguay C et al. 2014; Eton DT et al. 2012; Heid AR et al. 2020; Hardman R et al. 2021; Ørtenblad L et al. 2018; Löffler C et al. 2012; Morgan SA et al. 2019; O'Brien R et al. 2014; Gill A et al. 2014; Morris RL et al. 2011; Joensson ABR et al. 2020; Larkin J et al. 2021; Shin JW et al. 2022; Roberto KA et al. 2005; Sav A et al. 2013; Sand CD et al. 2021; Sells D et al. 2009; Rijken M et al. 2021; Porter T et al. 2020; van Merode T et al. 2018; White C et al. 2016; Zulman DM et al. 2015; Sun L et al. 2022; Townsend A et al. 2008; Ancker JS et al. 2015; Eton DT et al. 2015; Daker-White G et al. 2018; Etkind SN et al. 2022; Richardson LM et al. 2016; Ploeg J et al. 2017; Ploeg J et al. 2019; Slightam CA et al. 2018; |
| 7 | Financial work as a theme of burden | High confidence | No/Very minor concerns regarding methodological limitations, No/Very minor concerns regarding coherence, No/Very minor concerns regarding adequacy, and Minor concerns regarding relevance | Bayliss EA et al. 2003; Bissenbakker K et al. 2022; Åberg C et al. 2020; Clarke LH & Bennett EV 2013; Bardach SH et al. 2011; Favarato MH et al. 2021; Francis H et al. 2020; Coventry PA et al. 2015; Duguay C et al. 2014; Eton DT et al. 2012; Hardman R et al. 2021; Ørtenblad L et al. 2018; Morgan SA et al. 2019; O'Brien R et al. 2014; Morris RL et al. 2011; Larkin J et al. 2021; Shin JW et al. 2022; Roberto KA et al. 2005; Sav A et al. 2013; Sand CD et al. 2021; Sells D et al. 2009; van Merode T et al. 2018; White C et al. 2016; Zulman DM et al. 2015; Sun L et al. 2022; Townsend A et al. 2008; Ancker JS et al. 2015; Eton DT et al. 2015; Richardson LM et al. 2016; Ploeg J et al. 2017; Ploeg J et al. 2019; |
| 8 | Learning and adapting work as a theme of burden | High confidence | No/Very minor concerns regarding methodological limitations, No/Very minor concerns regarding coherence, No/Very minor concerns regarding adequacy, and Minor concerns regarding relevance | Bayliss EA et al. 2003; Clarke LH et al. 2008; Cheng C et al. 2019; Bower P et al. 2012; Bissenbakker K et al. 2022; Åberg C et al. 2020; Clarke LH & Bennett EV 2013; Clarke LH & Bennett E 2013; Bardach SH et al. 2011; Favarato MH et al. 2021; Francis H et al. 2020; Eckerblad J et al. 2015; Eckerblad J et al. 2020; Coventry PA et al. 2015; Duguay C et al. 2014; Eton DT et al. 2012; Heid AR et al. 2020; Hardman R et al. 2021; Ørtenblad L et al. 2018; Löffler C et al. 2012; Morgan SA et al. 2019; O'Brien R et al. 2014; Gill A et al. 2014; Morris RL et al. 2011; Joensson ABR et al. 2020; Larkin J et al. 2021; Shin JW et al. 2022; Roberto KA et al. 2005; Sav A et al. 2013; Sand CD et al. 2021; Sells D et al. 2009; Rijken M et al. 2021; Porter T et al. 2020; van Merode T et al. 2018; White C et al. 2016; Zulman DM et al. 2015; Sun L et al. 2022; Townsend A et al. 2008; Ancker JS et al. 2015; Eton DT et al. 2015; Daker-White G et al. 2018; Etkind SN et al. 2022; Richardson LM et al. 2016; Ploeg J et al. 2017; Ploeg J et al. 2019; Slightam CA et al. 2018; |
| 9 | Accumulation and complexity work as a theme of burden | High confidence | No/Very minor concerns regarding methodological limitations, No/Very minor concerns regarding coherence, No/Very minor concerns regarding adequacy, and No/Very minor concerns regarding relevance | Bayliss EA et al. 2003; Clarke LH et al. 2008; Cheng C et al. 2019; Bower P et al. 2012; Bissenbakker K et al. 2022; Åberg C et al. 2020; Clarke LH & Bennett EV 2013; Clarke LH & Bennett E 2013; Bardach SH et al. 2011; Favarato MH et al. 2021; Francis H et al. 2020; Eckerblad J et al. 2015; Eckerblad J et al. 2020; Coventry PA et al. 2015; Duguay C et al. 2014; Eton DT et al. 2012; Heid AR et al. 2020; Hardman R et al. 2021; Ørtenblad L et al. 2018; Löffler C et al. 2012; Morgan SA et al. 2019; O'Brien R et al. 2014; Gill A et al. 2014; Morris RL et al. 2011; Joensson ABR et al. 2020; Larkin J et al. 2021; Shin JW et al. 2022; Roberto KA et al. 2005; Sav A et al. 2013; Sand CD et al. 2021; Sells D et al. 2009; Rijken M et al. 2021; Porter T et al. 2020; van Merode T et al. 2018; White C et al. 2016; Zulman DM et al. 2015; Sun L et al. 2022; Townsend A et al. 2008; Ancker JS et al. 2015; Eton DT et al. 2015; Daker-White G et al. 2018; Etkind SN et al. 2022; Richardson LM et al. 2016; Ploeg J et al. 2017; Ploeg J et al. 2019; Slightam CA et al. 2018; |
| 10 | Biographical work - the impact of MLTCs on self-perception and life narrative | Moderate confidence | No/Very minor concerns regarding methodological limitations, No/Very minor concerns regarding coherence, Minor concerns regarding adequacy, and No/Very minor concerns regarding relevance | Clarke LH et al. 2008; Bower P et al. 2012; Clarke LH & Bennett E 2013; Francis H et al. 2020; Eckerblad J et al. 2020; Coventry PA et al. 2015; Heid AR et al. 2020; Hardman R et al. 2021; O'Brien R et al. 2014; Morris RL et al. 2011; Shin JW et al. 2022; Roberto KA et al. 2005; Sand CD et al. 2021; Porter T et al. 2020; Etkind SN et al. 2022; Slightam CA et al. 2018; |
| 11 | The impact of having MLTCs on time, including the time lost to healthcare activities, lacking time for medical interaction, time spent on administrative activities, time undertaking self-care, balancing with other activities such as work | Moderate confidence | No/Very minor concerns regarding methodological limitations, No/Very minor concerns regarding coherence, Minor concerns regarding adequacy, and No/Very minor concerns regarding relevance | Clarke LH & Bennett EV 2013; Bardach SH et al. 2011; Francis H et al. 2020; Duguay C et al. 2014; Eton DT et al. 2012; Ørtenblad L et al. 2018; Sav A et al. 2013; Sells D et al. 2009; van Merode T et al. 2018; White C et al. 2016; Zulman DM et al. 2015; Ancker JS et al. 2015; Eton DT et al. 2015; Daker-White G et al. 2018; Richardson LM et al. 2016; Ploeg J et al. 2019; |

**Evidence Profile Table**

| **#** | **Summarised review finding** | **Methodological limitations** | **Coherence** | **Adequacy** | **Relevance** | **GRADE-CERQual assessment of confidence** | **References** |
| --- | --- | --- | --- | --- | --- | --- | --- |
| 1 | People living with MLTCs do not just experience one type of work, but multiple, and these occur in differing combinations depending on the nature and combination of conditions and other factors. | No/Very minor concerns  **Explanation:** In general study quality was good. A common methodological weakness was that the relationship between researcher and participants did not appear to have been adequately considered. | No/Very minor concerns  **Explanation:** | No/Very minor concerns  **Explanation:** | No/Very minor concerns  **Explanation:** Only one study (Rijken) focused almost exclusively on one work theme. All other studies described the multifaceted nature of work for people with MLTCs. | High confidence  **Explanation:** No/Very minor concerns regarding methodological limitations, No/Very minor concerns regarding coherence, No/Very minor concerns regarding adequacy, and No/Very minor concerns regarding relevance | Ancker JS et al. 2015; Bardach SH et al. 2011; Bayliss EA et al. 2003; Bissenbakker K et al. 2022; Bower P et al. 2012; Cheng C et al. 2019; Clarke LH & Bennett E 2013; Clarke LH & Bennett EV 2013; Clarke LH et al. 2008; Coventry PA et al. 2015; Daker-White G et al. 2018; Duguay C et al. 2014; Eckerblad J et al. 2015; Eckerblad J et al. 2020; Etkind SN et al. 2022; Eton DT et al. 2012; Eton DT et al. 2015; Favarato MH et al. 2021; Francis H et al. 2020; Gill A et al. 2014; Hardman R et al. 2021; Heid AR et al. 2020; Joensson ABR et al. 2020; Larkin J et al. 2021; Löffler C et al. 2012; Morgan SA et al. 2019; Morris RL et al. 2011; O'Brien R et al. 2014; Ploeg J et al. 2017; Ploeg J et al. 2019; Porter T et al. 2020; Richardson LM et al. 2016; Roberto KA et al. 2005; Sand CD et al. 2021; Sav A et al. 2013; Sells D et al. 2009; Shin JW et al. 2022; Slightam CA et al. 2018; Sun L et al. 2022; Townsend A et al. 2008; White C et al. 2016; Zulman DM et al. 2015; van Merode T et al. 2018; Åberg C et al. 2020; Ørtenblad L et al. 2018; |
| 2 | Symptom work as a theme of burden | No/Very minor concerns  **Explanation:** In general study quality was good. A common methodological weakness (in 41/46 studies) was that the relationship between researcher and participants did not appear to have been adequately considered. | No/Very minor concerns  **Explanation:**  Symptoms acting as burden/work was a common finding across all studies. Although there was variation in the specific symptoms experienced, depending on the study populations, the finding reflects the complexity and variation of the data. | No/Very minor concerns  **Explanation:** | No/Very minor concerns  **Explanation:** | High confidence  **Explanation:** No/Very minor concerns regarding methodological limitations, No/Very minor concerns regarding coherence, No/Very minor concerns regarding adequacy, and No/Very minor concerns regarding relevance | Ancker JS et al. 2015; Bardach SH et al. 2011; Bayliss EA et al. 2003; Bissenbakker K et al. 2022; Bower P et al. 2012; Cheng C et al. 2019; Clarke LH & Bennett E 2013; Clarke LH & Bennett EV 2013; Clarke LH et al. 2008; Coventry PA et al. 2015; Daker-White G et al. 2018; Duguay C et al. 2014; Eckerblad J et al. 2015; Eckerblad J et al. 2020; Etkind SN et al. 2022; Eton DT et al. 2012; Eton DT et al. 2015; Favarato MH et al. 2021; Francis H et al. 2020; Gill A et al. 2014; Hardman R et al. 2021; Heid AR et al. 2020; Joensson ABR et al. 2020; Larkin J et al. 2021; Löffler C et al. 2012; Morgan SA et al. 2019; Morris RL et al. 2011; O'Brien R et al. 2014; Ploeg J et al. 2017; Ploeg J et al. 2019; Porter T et al. 2020; Richardson LM et al. 2016; Rijken M et al. 2021; Roberto KA et al. 2005; Sand CD et al. 2021; Sav A et al. 2013; Sells D et al. 2009; Shin JW et al. 2022; Slightam CA et al. 2018; Sun L et al. 2022; Townsend A et al. 2008; White C et al. 2016; Zulman DM et al. 2015; van Merode T et al. 2018; Åberg C et al. 2020; Ørtenblad L et al. 2018; |
| 3 | Emotional work as a theme of burden | No/Very minor concerns  **Explanation:** In general study quality was good. A common methodological weakness (in 41/46 studies) was that the relationship between researcher and participants did not appear to have been adequately considered. | No/Very minor concerns  **Explanation:** Emotions were described as a component of the challenges of living with MLTCs in all studies. There was variation in the specific emotions expressed and the extent to which they affected the study participants but dealing with the emotional impact of MLTCs was a very common finding. | No/Very minor concerns  **Explanation:** As a ubiquitous finding of all studies we have no concerns about adequacy | No/Very minor concerns  **Explanation:** | High confidence  **Explanation:** No/Very minor concerns regarding methodological limitations, No/Very minor concerns regarding coherence, No/Very minor concerns regarding adequacy, and No/Very minor concerns regarding relevance | Ancker JS et al. 2015; Bardach SH et al. 2011; Bayliss EA et al. 2003; Bissenbakker K et al. 2022; Bower P et al. 2012; Cheng C et al. 2019; Clarke LH & Bennett E 2013; Clarke LH & Bennett EV 2013; Clarke LH et al. 2008; Coventry PA et al. 2015; Daker-White G et al. 2018; Duguay C et al. 2014; Eckerblad J et al. 2015; Eckerblad J et al. 2020; Etkind SN et al. 2022; Eton DT et al. 2012; Eton DT et al. 2015; Favarato MH et al. 2021; Francis H et al. 2020; Gill A et al. 2014; Hardman R et al. 2021; Heid AR et al. 2020; Joensson ABR et al. 2020; Larkin J et al. 2021; Löffler C et al. 2012; Morgan SA et al. 2019; Morris RL et al. 2011; O'Brien R et al. 2014; Ploeg J et al. 2017; Ploeg J et al. 2019; Porter T et al. 2020; Richardson LM et al. 2016; Rijken M et al. 2021; Roberto KA et al. 2005; Sand CD et al. 2021; Sav A et al. 2013; Sells D et al. 2009; Shin JW et al. 2022; Slightam CA et al. 2018; Sun L et al. 2022; Townsend A et al. 2008; White C et al. 2016; Zulman DM et al. 2015; van Merode T et al. 2018; Åberg C et al. 2020; Ørtenblad L et al. 2018; |
| 4 | Investigation and monitoring work as a theme of burden | No/Very minor concerns  **Explanation:** In general study quality was good. A common methodological weakness was that the relationship between researcher and participants did not appear to have been adequately considered. | No/Very minor concerns  **Explanation:** The work associated with medical investigations and/or monitoring of health conditions, including managing personal health information, was a finding across many of the studies (30/46). The specific investigations and monitoring tasks varied between studies but this was a reflection of the diversity of study populations. | No/Very minor concerns  **Explanation:** | No/Very minor concerns  **Explanation:** While study populations and settings varied quite widely, the work of investigation and monitoring was a common theme. | High confidence  **Explanation:** No/Very minor concerns regarding methodological limitations, No/Very minor concerns regarding coherence, No/Very minor concerns regarding adequacy, and No/Very minor concerns regarding relevance | Ancker JS et al. 2015; Bardach SH et al. 2011; Bayliss EA et al. 2003; Bower P et al. 2012; Cheng C et al. 2019; Clarke LH & Bennett EV 2013; Coventry PA et al. 2015; Daker-White G et al. 2018; Eckerblad J et al. 2020; Etkind SN et al. 2022; Eton DT et al. 2012; Eton DT et al. 2015; Favarato MH et al. 2021; Gill A et al. 2014; Hardman R et al. 2021; Joensson ABR et al. 2020; Larkin J et al. 2021; Löffler C et al. 2012; Morgan SA et al. 2019; Morris RL et al. 2011; Ploeg J et al. 2017; Porter T et al. 2020; Richardson LM et al. 2016; Rijken M et al. 2021; Shin JW et al. 2022; Slightam CA et al. 2018; Townsend A et al. 2008; White C et al. 2016; Zulman DM et al. 2015; Ørtenblad L et al. 2018; |
| 5 | Health service and administration work as a theme of burden | No/Very minor concerns  **Explanation:** In general study quality was good. A common methodological weakness was that the relationship between researcher and participants did not appear to have been adequately considered. | No/Very minor concerns  **Explanation:** Health service and administration work was a common finding across studies. For example, the work associated with booking multiple appointments, communicating and travel were represented in many studies. | No/Very minor concerns  **Explanation:** | No/Very minor concerns  **Explanation:** | High confidence  **Explanation:** No/Very minor concerns regarding methodological limitations, No/Very minor concerns regarding coherence, No/Very minor concerns regarding adequacy, and No/Very minor concerns regarding relevance | Ancker JS et al. 2015; Bardach SH et al. 2011; Bayliss EA et al. 2003; Bissenbakker K et al. 2022; Bower P et al. 2012; Cheng C et al. 2019; Clarke LH & Bennett E 2013; Clarke LH & Bennett EV 2013; Clarke LH et al. 2008; Coventry PA et al. 2015; Daker-White G et al. 2018; Duguay C et al. 2014; Eckerblad J et al. 2015; Eckerblad J et al. 2020; Etkind SN et al. 2022; Eton DT et al. 2012; Eton DT et al. 2015; Favarato MH et al. 2021; Francis H et al. 2020; Gill A et al. 2014; Hardman R et al. 2021; Heid AR et al. 2020; Joensson ABR et al. 2020; Larkin J et al. 2021; Löffler C et al. 2012; Morgan SA et al. 2019; Morris RL et al. 2011; O'Brien R et al. 2014; Ploeg J et al. 2017; Ploeg J et al. 2019; Porter T et al. 2020; Richardson LM et al. 2016; Rijken M et al. 2021; Roberto KA et al. 2005; Sand CD et al. 2021; Sav A et al. 2013; Sells D et al. 2009; Shin JW et al. 2022; Slightam CA et al. 2018; Sun L et al. 2022; Townsend A et al. 2008; van Merode T et al. 2018; Åberg C et al. 2020; Ørtenblad L et al. 2018; |
| 6 | Medication work as a theme of burden | No/Very minor concerns  **Explanation:** In general study quality was good. A common methodological weakness (in 41/46 studies) was that the relationship between researcher and participants did not appear to have been adequately considered. | No/Very minor concerns  **Explanation:** Work associated with managing medications or their effects was a common theme across all studies. Some studies also reported positive aspects of taking medication highlighted by some participants. | No/Very minor concerns  **Explanation:** | No/Very minor concerns  **Explanation:** | High confidence  **Explanation:** No/Very minor concerns regarding methodological limitations, No/Very minor concerns regarding coherence, No/Very minor concerns regarding adequacy, and No/Very minor concerns regarding relevance | Ancker JS et al. 2015; Bardach SH et al. 2011; Bayliss EA et al. 2003; Bissenbakker K et al. 2022; Bower P et al. 2012; Cheng C et al. 2019; Clarke LH & Bennett E 2013; Clarke LH & Bennett EV 2013; Clarke LH et al. 2008; Coventry PA et al. 2015; Daker-White G et al. 2018; Duguay C et al. 2014; Eckerblad J et al. 2015; Eckerblad J et al. 2020; Etkind SN et al. 2022; Eton DT et al. 2012; Eton DT et al. 2015; Favarato MH et al. 2021; Francis H et al. 2020; Gill A et al. 2014; Hardman R et al. 2021; Heid AR et al. 2020; Joensson ABR et al. 2020; Larkin J et al. 2021; Löffler C et al. 2012; Morgan SA et al. 2019; Morris RL et al. 2011; O'Brien R et al. 2014; Ploeg J et al. 2017; Ploeg J et al. 2019; Porter T et al. 2020; Richardson LM et al. 2016; Rijken M et al. 2021; Roberto KA et al. 2005; Sand CD et al. 2021; Sav A et al. 2013; Sells D et al. 2009; Shin JW et al. 2022; Slightam CA et al. 2018; Sun L et al. 2022; Townsend A et al. 2008; White C et al. 2016; Zulman DM et al. 2015; van Merode T et al. 2018; Åberg C et al. 2020; Ørtenblad L et al. 2018; |
| 7 | Financial work as a theme of burden | No/Very minor concerns  **Explanation:** In general study quality was good. A common methodological weakness was that the relationship between researcher and participants did not appear to have been adequately considered. | No/Very minor concerns  **Explanation:** | No/Very minor concerns  **Explanation:** | Minor concerns  **Explanation:** Minor concerns regarding relevance because of the variation in study populations, geographical locations and health and social care systems represented in the different studies. | High confidence  **Explanation:** No/Very minor concerns regarding methodological limitations, No/Very minor concerns regarding coherence, No/Very minor concerns regarding adequacy, and Minor concerns regarding relevance | Ancker JS et al. 2015; Bardach SH et al. 2011; Bayliss EA et al. 2003; Bissenbakker K et al. 2022; Clarke LH & Bennett EV 2013; Coventry PA et al. 2015; Duguay C et al. 2014; Eton DT et al. 2012; Eton DT et al. 2015; Favarato MH et al. 2021; Francis H et al. 2020; Hardman R et al. 2021; Larkin J et al. 2021; Morgan SA et al. 2019; Morris RL et al. 2011; O'Brien R et al. 2014; Ploeg J et al. 2017; Ploeg J et al. 2019; Richardson LM et al. 2016; Roberto KA et al. 2005; Sand CD et al. 2021; Sav A et al. 2013; Sells D et al. 2009; Shin JW et al. 2022; Sun L et al. 2022; Townsend A et al. 2008; White C et al. 2016; Zulman DM et al. 2015; van Merode T et al. 2018; Åberg C et al. 2020; Ørtenblad L et al. 2018; |
| 8 | Learning and adapting work as a theme of burden | No/Very minor concerns  **Explanation:** In general study quality was good. A common methodological weakness (in 41/46 studies) was that the relationship between researcher and participants did not appear to have been adequately considered. | No/Very minor concerns  **Explanation:** The need to adapt aspects of life to manage MLTCs and to learn new skills such as those required to support self-management were a very common finding | No/Very minor concerns  **Explanation:** | Minor concerns  **Explanation:** Minor concerns regarding relevance because the need to adapt and learn varied by factors such as the specific long-term conditions being considered and the differing characteristics of the study participants (e.g. age, gender, socioeconomic status). | High confidence  **Explanation:** No/Very minor concerns regarding methodological limitations, No/Very minor concerns regarding coherence, No/Very minor concerns regarding adequacy, and Minor concerns regarding relevance | Ancker JS et al. 2015; Bardach SH et al. 2011; Bayliss EA et al. 2003; Bissenbakker K et al. 2022; Bower P et al. 2012; Cheng C et al. 2019; Clarke LH & Bennett E 2013; Clarke LH & Bennett EV 2013; Clarke LH et al. 2008; Coventry PA et al. 2015; Daker-White G et al. 2018; Duguay C et al. 2014; Eckerblad J et al. 2015; Eckerblad J et al. 2020; Etkind SN et al. 2022; Eton DT et al. 2012; Eton DT et al. 2015; Favarato MH et al. 2021; Francis H et al. 2020; Gill A et al. 2014; Hardman R et al. 2021; Heid AR et al. 2020; Joensson ABR et al. 2020; Larkin J et al. 2021; Löffler C et al. 2012; Morgan SA et al. 2019; Morris RL et al. 2011; O'Brien R et al. 2014; Ploeg J et al. 2017; Ploeg J et al. 2019; Porter T et al. 2020; Richardson LM et al. 2016; Rijken M et al. 2021; Roberto KA et al. 2005; Sand CD et al. 2021; Sav A et al. 2013; Sells D et al. 2009; Shin JW et al. 2022; Slightam CA et al. 2018; Sun L et al. 2022; Townsend A et al. 2008; White C et al. 2016; Zulman DM et al. 2015; van Merode T et al. 2018; Åberg C et al. 2020; Ørtenblad L et al. 2018; |
| 9 | Accumulation and complexity work as a theme of burden | No/Very minor concerns  **Explanation:** In general study quality was good. A common methodological weakness (in 41/46 studies) was that the relationship between researcher and participants did not appear to have been adequately considered. | No/Very minor concerns  **Explanation:** | No/Very minor concerns  **Explanation:** | No/Very minor concerns  **Explanation:** | High confidence  **Explanation:** No/Very minor concerns regarding methodological limitations, No/Very minor concerns regarding coherence, No/Very minor concerns regarding adequacy, and No/Very minor concerns regarding relevance | Ancker JS et al. 2015; Bardach SH et al. 2011; Bayliss EA et al. 2003; Bissenbakker K et al. 2022; Bower P et al. 2012; Cheng C et al. 2019; Clarke LH & Bennett E 2013; Clarke LH & Bennett EV 2013; Clarke LH et al. 2008; Coventry PA et al. 2015; Daker-White G et al. 2018; Duguay C et al. 2014; Eckerblad J et al. 2015; Eckerblad J et al. 2020; Etkind SN et al. 2022; Eton DT et al. 2012; Eton DT et al. 2015; Favarato MH et al. 2021; Francis H et al. 2020; Gill A et al. 2014; Hardman R et al. 2021; Heid AR et al. 2020; Joensson ABR et al. 2020; Larkin J et al. 2021; Löffler C et al. 2012; Morgan SA et al. 2019; Morris RL et al. 2011; O'Brien R et al. 2014; Ploeg J et al. 2017; Ploeg J et al. 2019; Porter T et al. 2020; Richardson LM et al. 2016; Rijken M et al. 2021; Roberto KA et al. 2005; Sand CD et al. 2021; Sav A et al. 2013; Sells D et al. 2009; Shin JW et al. 2022; Slightam CA et al. 2018; Sun L et al. 2022; Townsend A et al. 2008; White C et al. 2016; Zulman DM et al. 2015; van Merode T et al. 2018; Åberg C et al. 2020; Ørtenblad L et al. 2018; |
| 10 | Biographical work - the impact of MLTCs on self-perception and life narrative | No/Very minor concerns  **Explanation:** In general study quality was good. A common methodological weakness was that the relationship between researcher and participants did not appear to have been adequately considered. | No/Very minor concerns  **Explanation:** | Minor concerns  **Explanation:** Minor concerns regarding adequacy because this phenomenon did not arise in all studies. | No/Very minor concerns  **Explanation:** | Moderate confidence  **Explanation:** No/Very minor concerns regarding methodological limitations, No/Very minor concerns regarding coherence, Minor concerns regarding adequacy, and No/Very minor concerns regarding relevance | Bower P et al. 2012; Clarke LH & Bennett E 2013; Clarke LH et al. 2008; Coventry PA et al. 2015; Eckerblad J et al. 2020; Etkind SN et al. 2022; Francis H et al. 2020; Hardman R et al. 2021; Heid AR et al. 2020; Morris RL et al. 2011; O'Brien R et al. 2014; Porter T et al. 2020; Roberto KA et al. 2005; Sand CD et al. 2021; Shin JW et al. 2022; Slightam CA et al. 2018; |
| 11 | The impact of having MLTCs on time, including the time lost to healthcare activities, lacking time for medical interaction, time spent on administrative activities, time undertaking self-care, balancing with other activities such as work | No/Very minor concerns  **Explanation:** In general study quality was good | No/Very minor concerns  **Explanation:** | Minor concerns  **Explanation:** Minor concerns regarding adequacy because of this aspect only being reflected in some of the studies | No/Very minor concerns  **Explanation:** | Moderate confidence  **Explanation:** No/Very minor concerns regarding methodological limitations, No/Very minor concerns regarding coherence, Minor concerns regarding adequacy, and No/Very minor concerns regarding relevance | Ancker JS et al. 2015; Bardach SH et al. 2011; Clarke LH & Bennett EV 2013; Daker-White G et al. 2018; Duguay C et al. 2014; Eton DT et al. 2012; Eton DT et al. 2015; Francis H et al. 2020; Ploeg J et al. 2019; Richardson LM et al. 2016; Sav A et al. 2013; Sells D et al. 2009; White C et al. 2016; Zulman DM et al. 2015; van Merode T et al. 2018; Ørtenblad L et al. 2018; |

**Supplementary Table 4 - GRIPP2 MELD-B Qualitative Evidence Synthesis (QES)**

Patient and public involvement with this study

Table S6: GRIPP2 reporting checklist (short form)

| Section and topic | Item | Reported on page number |
| --- | --- | --- |
| 1: Aim | Report the aim of PPI in the study | 140 |
| 2: Methods | Provide a clear description of the methods used for PPI in the study | 140 |
| 3: Study results | Outcomes—Report the results of PPI in the study, including both positive and negative outcomes | 141 |
| 4: Discussion and conclusions | Outcomes—Comment on the extent to which PPI influenced the study overall. Describe positive and negative effects | 141-2 |
| 5: Reflections/critical perspective | Comment critically on the study, reflecting on the things that went well and those that did not, so others can learn from this experience | 142 |

**Aim**

The aim of Patient and Public Involvement (PPI) in the Qualitative Evidence Synthesis (QES) for the MELD B project was to:

1. Incorporate the views, perspectives and experiences of those living with or caring for those living with multiple long-term conditions (MLTCs)
2. Understand the perspective of people living with MLTCs on the concept of burden including how it should be characterised and described, and whether burden is the correct term to use
3. Address the questions ‘What are the burdens associated with managing MLTCs? ’and ‘Are they likely to be captured adequately in routine health data?’
4. Address the questions ‘What health conditions and diseases are particularly associated with burden?’ and ‘What symptoms are particularly burdensome?’
5. Help use the findings from the QES to formulate a subsequent study, likely a Delphi consensus study, to understand what aspects are most burdensome.

**Methods**

We used a mix of methods to incorporate PPI in the QES. 3 members of the MELD B PPI Advisory Board were specifically associated with this aspect of the MELB B project and attended all the research meetings. Other members of the MELD B PPI Advisory Board were involved on an ad hoc basis on specific parts of the research. As well as the PPI Advisory Board, an additional PPI group composed of women living with MLTCs in the context of severe and enduring socioeconomic disadvantage discussed aspects of the research.

We made time for reflexive discussions around the whole concept of “burden” and acknowledged how our positionality, perspectives and lived experience affected our thinking. This led to us questioning whether burden is the right term to use and exploring the concept of work, building on the Corbin and Strauss research. We have reflected that active PPI involvement in all the discussions led to the development of our thinking in a way that might not have been achieved if the discussions were limited to the researchers.

Methods used included

1. Helping to identify search terms for the QES
2. Discussing and analysing the findings from the included papers
3. Helping to plan the Delphi including what methods should be used to facilitate it
4. Co-authoring this paper
5. Including insights from the PPI in the reporting of the research progress to the funder.

One member of the PPI group read the final 46 papers looking for, and synthesising, the evidence of PPI in the research and presented the findings to the team. The results from this are included in the paper.

We also invited members of the MELD-B PPI Advisory Board to a meeting where the concept of burden proxies were discussed, including what is the burden from an individual’s perspective. Symptoms such as pain and stress were felt to be proxy burdens. The main finding from the meeting was that burdensomeness is a poor term which doesn’t adequately capture the impact and complexity involved in living with MLTCs, including understanding an individual’s personal context. A PPI member facilitated the meeting, synthesised the findings and shared them with the attendees for comment.

One of the public contributor co-authors of this paper wrote the first draft of the GRIPP2 and circulated it to interested members of the research team to add comments and suggestions.

**Study results**

Public contributors involved in the QES reported feeling “like part of the research team” and appreciated the opportunity to get involved in different parts of the QES which related to their skills and interests.

People were offered payment for their time at NIHR rates, meetings were well facilitated, organised in advance and notes were circulated afterwards. Having a dedicated PPI officer to contact and manage the involvement was appreciated and crucial to the success.

Time was made for challenging discussions around the whole concept of burden and burdensomeness, including the appropriateness of the term. The emotional labour of living with MLTCs and being involved in the research was acknowledged.

A QES is a technical endeavour and it can be challenging to find ways to meaningfully involve people in all aspects of the research. Although we made time for meetings and conversations there were specific deadlines to be met, as always with involvement there is a tension between pragmatism and perfection.

**Discussion and Conclusions**

The openness of the research team to discussing the whole concept of burden with regards to MLTCs, including if this is an appropriate term to use, was critical. The whole research team agreed that PPI has substantially influenced and improved the quality of the work undertaken in this evidence synthesis. We had many discussions about whose burden is it anyway, what is the burden and who should get to define it. Early discussions also centred on the impact of living with MLTCs which helped shape much of our thinking. We also spent a lot of time discussing the concept of work, as opposed to burden, and whether the likes of emotions and symptoms were work or subjective experiences. Members of the PPI team utilised their experience of living with MLTCs to emphasise the importance of context and understanding each individual’s experience, not treating people as a homogenous mass. Also, that the work of living with MLTCs doesn’t remain static, the workload increases and decreases over time.

We also discussed how work, or burden, is poorly captured in routine health data which isn’t collected from the patient’s point of view and acknowledged that it is often written by members of clinical teams working under significant time pressure. We were shocked that out of the 46 papers in the final review only 5 of them had any PPI in the research, leading to us questioning who is setting the research agenda when it comes to qualitive research into the work of living with MLTCs. Are the results of the research useful if they don’t reflect the priorities and topics of people living with MLTCs?

One of the public contributors involved with the research commented that they could see the conversations we have had as a team reflected in this paper, especially around replacing the concept of burden with work.

There is no doubt that it is challenging to embrace the “productive tensions”, be reflexive and understand that everyone in the team is coming from a different positionality and perspective. We didn’t always agree, conversations were often uncomfortable and could cause upset. But, we worked hard to overcome this, understand each other and make compromises. As an academic member of the team commented the end result is something that we should be proud of.

**Reflections/ critical perspective**

We feel that this paper shows that given the right conditions e.g. resources, time, understanding, reflexivity etc PPI can make an impact on research such as an evidence synthesis. Disagreement can be productive if coupled with respect and honesty. Making time for conversations is crucial as is acting on suggestions from PPI members, having the resources to remunerate them and a dedicated person to facilitate involvement.

PPI clearly improved the quality and depth of thinking and analysis in the evidence synthesis.
